# Electrocatalytic Energy Release of Norbornadiene‐Based Molecular Solar Thermal Systems: Tuning the Electrochemical Stability by Molecular Design

**DOI:** 10.1002/cssc.202201483

**Published:** 2022-11-11

**Authors:** Evanie Franz, Daniel Krappmann, Lukas Fromm, Tobias Luchs, Andreas Görling, Andreas Hirsch, Olaf Brummel, Jörg Libuda

**Affiliations:** ^1^ Interface Research and Catalysis Erlangen Center for Interface Research and Catalysis Friedrich-Alexander-Universität Erlangen-Nürnberg Egerlandstraße 3 91058 Erlangen Germany; ^2^ Chair of Organic Chemistry II Friedrich-Alexander-Universität Erlangen-Nürnberg Nikolaus-Fiebiger-Straße 10 91058 Erlangen Germany; ^3^ Lehrstuhl für Theoretische Chemie Friedrich-Alexander-Universität Erlangen-Nürnberg Egerlandstraße 3 91058 Erlangen Germany

**Keywords:** electrochemistry, energy storage, photochemistry, photoswitches, solar thermal fuels

## Abstract

Molecular solar thermal (MOST) systems, such as the norbornadiene/quadricyclane (NBD/QC) couple, combine solar energy conversion, storage, and release in a simple one‐photon one‐molecule process. Triggering the energy release electrochemically enables high control of the process, high selectivity, and reversibility. In this work, the influence of the molecular design of the MOST couple on the electrochemically triggered back‐conversion reaction was addressed for the first time. The MOST systems phenyl‐ethyl ester‐NBD/QC (NBD1/QC1) and *p*‐methoxyphenyl‐ethyl ester‐NBD/QC (NBD2/QC2) were investigated by in‐situ photoelectrochemical infrared spectroscopy, voltammetry, and density functional theory modelling. For QC1, partial decomposition (40 %) was observed upon back‐conversion and along with a voltammetric peak at 0.6 V_fc_, which was assigned primarily to decomposition. The back‐conversion of QC2, however, occurred without detectable side products, and the corresponding peak at 0.45 V_fc_ was weaker by a factor of 10. It was concluded that the electrochemical stability of a NBD/QC couple is easy tunable by simple structural changes. Furthermore, the charge input and, therefore, the current for the electrochemically triggered energy release is very low, which ensures a high overall efficiency of the MOST system.

## Graphical abstract

**Making the MOST of it**: Molecular solar thermal (MOST) systems combine solar energy conversion, storage, and release in a single molecule. For a properly designed norbornadiene/quadricyclane‐based MOST system, the energy release can be triggered by an electrochemically initiated radical chain reaction, which is highly selective and requires only minimal energy input.

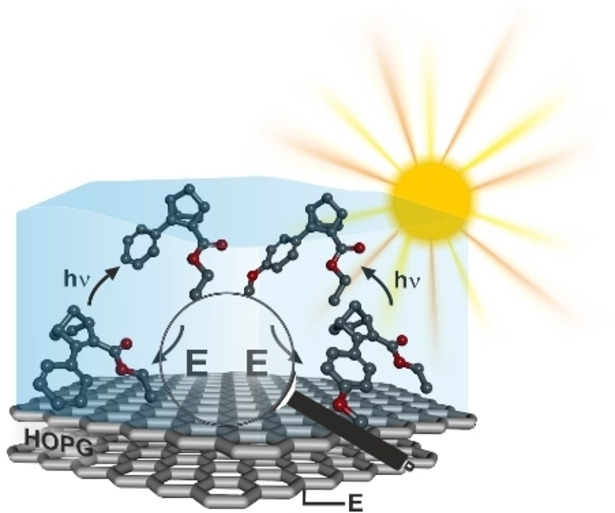

## Introduction

**Figure 1:**
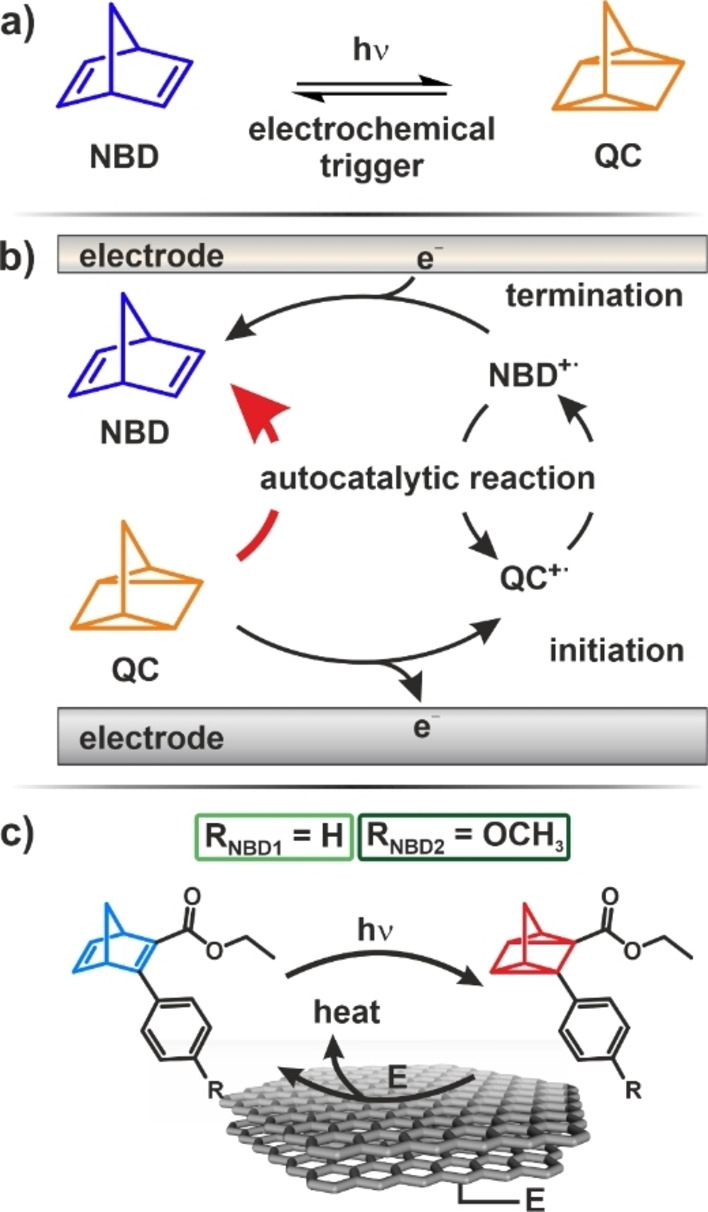
Simplified concept of the electrochemically triggered energy release in NBD/QC based MOST systems. (a) Reaction overview. (b) Proposed mechanism. (c) NBD/QC derivatives studied in this work. E=applied potential.

Beside the established methods for solar energy storage, such as photovoltaics/batteries or power‐to‐X, there is also a simple molecular approach based on so‐called molecular solar thermal systems (MOST).[^1^, ^2^] MOST systems combine energy conversion, storage, and release in a simple one‐photon one‐molecule process. They consist of compounds that form energy‐rich meta‐stable photo‐isomers upon irradiation and, at a later point, release this energy in form of heat.[^3^, ^4^, ^5^, ^6^] Examples of MOST systems are the (*E*)/(*Z*)‐azobenzene couple,[^7^, ^8^, ^9^, ^10^] the dihydroazulene/ vinylheptafulvene couple,^[11]^ and azaborine/BN‐dewar couple.^[12]^ One of the best‐studied MOST system is the norbornadiene/quadricyclane (NBD/QC) couple (Figure 1a). Here, NBD isomerizes via a [2+2] cycloaddition to quadricyclane (QC) upon absorption of UV light.[^13^, ^14^, ^15^, ^16^] However, the pristine NBD absorbs at wavelengths below 300 nm, which makes it necessary to use a photosensitizer.[^17^, ^18^] In order to overcome this limitation, a variety of NBD/QC derivatives were developed in the last years.^[1]^ In this context, a promising strategy is to functionalize the NBD derivatives at the positions 2 and 3 with electron‐withdrawing and electron‐donating substituents, respectively. This results in a so‐called push‐pull system to shift the absorption spectrum to the red.[^19^, ^20^, ^21^]

To develop applications based on MOST systems, it is essential to control the energy release at will. The energy release from MOST systems can, for example, be initiated thermally.[^20^, ^21^, ^22^, ^23^] However, this method lacks of controllability, and the fact that the triggering process consumes energy reduces the overall efficiency of the storage system. There are different ways to overcome these drawbacks, for example, molecular design[^24^, ^25^] or choosing an appropriate trigger to release the energy. Commonly used triggers are metal complex catalysts,[^26^, ^27^, ^28^, ^29^, ^30^, ^31^] copper(II) or tin(II) salts,^[32]^ or heterogeneous catalysts.[^33^, ^34^, ^35^, ^36^]

A particular intriguing concept is to initiate the heat release electrochemically.[^7^, ^37^, ^38^, ^39^, ^40^, ^41^] For the NBD/QC system, the electrochemically triggered back‐conversion occurs via a hole‐catalyzed (oxidative) reaction pathway. QC is oxidized to a QC^+^⋅ radical, and, subsequently, the QC^+^⋅ radical initiates a chain reaction, which catalyzes the back‐conversion. The proposed reaction mechanism is illustrated in Figure 1b. The applied potential controls the concentration of the QC^+^⋅ radicals and, consequently, the reaction rate of the back‐conversion.^[37]^ Note, however, that the overall release process is not a redox reaction.

The electrochemical pathway enables us to trigger the back‐conversion with very high selectivity^[42]^ and reversibility.[^38^, ^39^] In a recent study we achieved a reversibility of 99.8 % using an inert electrode material and a tailor‐made NBD/QC couple [2‐cyano‐3‐(3,4dimethoxyphenyl)‐NBD/QC (NBD’/QC’)] with push‐pull functionalization of the motif.^[39]^ However, the influence of the push‐pull system on the electrochemical stability has not been studied in more detail so far. In the present study, we address this very important aspect for the first time. We combined electrochemical, spectroscopic, and modelling methods to study NBD derivatives with different push‐pull functionalization. We choose an NBD derivative, with different acceptor, which reaches *t*_1/2_ values and absorption properties similar to NBD’/QC’.^[19]^ In specific, we investigate the energy release in the MOST systems phenyl‐ethyl ester‐norbornadiene/quadricyclane (NBD1/QC1) and *p*‐methoxyphenyl‐ethyl ester‐norbornadiene/quadricyclane (NBD2/QC2) (see Figure 1c). We demonstrate that the methoxy functionality strongly affects the electrochemical stability of the MOST system. In addition, we show that in a properly designed MOST system, very low concentrations of radical species are sufficient to trigger the energy release quantitatively. As a result, the additional energy input, which is necessary to trigger the energy release, is very small.

## Results and Discussion

In a first step, we studied the oxidative reaction channel for the two different QC derivatives with density functional theory (DFT) calculations using the Perdew‐Burke‐Ernzerhof (PBE) functional.^[43]^ To assess the relevant electrochemical properties for the energy release reaction, we calculated the oxidation potentials of the geometry optimized neutral and cationic species in the gas phase. Additionally, we included solvent effects with the COSMO solvation model for a dielectric constant of 37.5, corresponding to the value of acetonitrile (MeCN, see the Experimental Section for details). Note that MeCN was used as a solvent in the experiments described below. To test the dependence of the results on the choice of the employed density functional we repeated the calculations with the B3LYP functional (see Figure S10).^[44]^ Only small changes were observed when changing the density functional. In particular, the calculated ionization potentials remain virtually unchanged.

**Figure 2:**
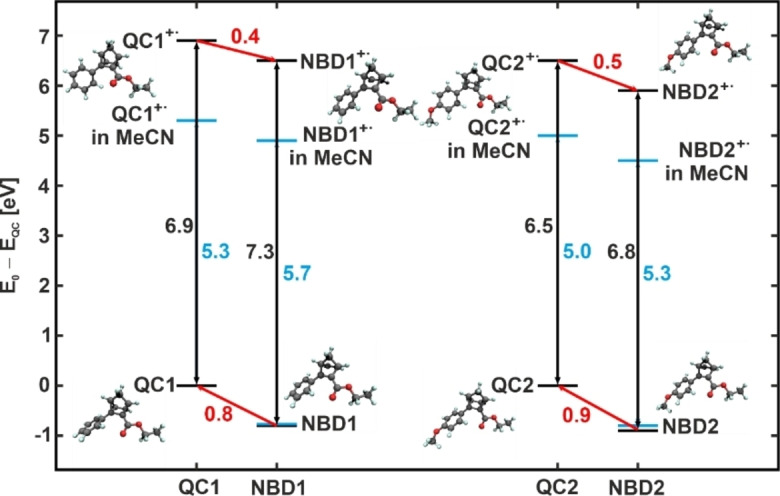
Energies of the considered QC, QC^+^⋅, NBD^+^⋅, and NBD compounds relative to QC for the derivatives NBD1/QC1 and NBD2/QC2 as derived from DFT calculations with PBE exchange‐correlation functional. Black: calculations in gas phase; blue: calculations taking into account the COSMO solvent model.

The corresponding energies of the considered QC, QC^+^⋅, NBD^+^⋅, and NBD compounds relative to the QC molecule are depicted in Figure 2. For QC1 (left), we obtained an ionization energy of 6.9 eV (formation of the radical cation), while the ionization energy decreases to 6.5 eV for QC2 (right). A similar effect is observed for the NBD isomers. The decrease in ionization energy for NBD2/QC2 is attributed to the influence of the methoxy group, which strengthens the electron donating properties of one moiety and thereby stabilizes the ionized state. In addition, we observe a stabilizing effect of the solvent as demonstrated by the calculations with the COSMO model (bright blue bars in Figure 2). Including the dielectric medium leads to a stabilization of charge, which reduces the energy of the cations.

The observed trends in ionization energies correspond to the eigenvalues of the highest occupied molecular orbitals (HOMOs). Figure S11 shows the eigenvalues of the four energetically highest occupied and the two energetically lowest unoccupied orbitals of NBD1, QC1, NBD2, and QC2, as well as contour plots of the orbitals calculated with the PBE functional including the COSMO model. Formally, the negative of the HOMO eigenvalue equals the ionization energy in Kohn‐Sham DFT. For semilocal functionals like the PBE functional used here, the HOMO eigenvalue typically is too high by about 1 or 2 eV. This can be observed here as well by comparing Figure 2 and Figure S10. The relative positions of the HOMO eigenvalues, however, match the trends of the ionization energies. The eigenvalues of the HOMOs of NBD1 and NBD2 are lower than the respective HOMO eigenvalues of QC1 and QC2 consistent with a higher ionization energy of the NBD compounds compared to the QC ones. Moreover, the eigenvalues of the HOMOs of NBD2 and QC2 are higher than those of NBD1 and QC1, which reflects the lower ionization potentials of NBD2 and QC2 compared to NBD1 and QC1.

**Figure 3:**
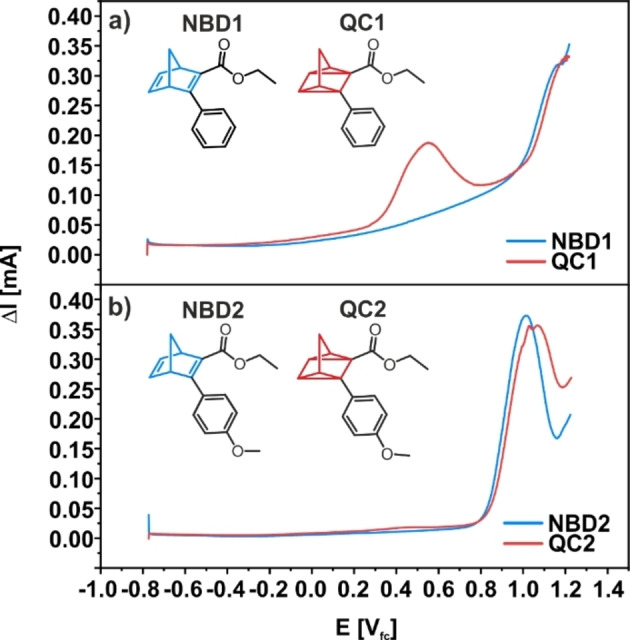
Electrochemical characterization of NBD1 and NBD2. (a) Differential pulse voltammograms of NBD1 (blue) and NBD1 after 3 h of irradiation (red) in MeCN. (b) Differential pulse voltammogram of NBD2 (blue) and NBD2 after 3 h of irradiation (red) in MeCN. Parameters: step size: 2 mV, sample period: 0.5 s, pulse size: 20 mV, pulse time: 0.25 s.

In the next step, we investigated the electrochemical response of NBD1 and NBD2 and their QC isomers by voltammetric methods. In specific, we used differential pulse voltammetry (DPV, Figure 3) and cyclic voltammetry (CV, see the Supporting Information). For both NBD derivatives we used 10 mm solutions in MeCN. The supporting electrolyte was 0.1 m tetrabutylammonium perchlorate (TBAP), and graphite was employed as an inert working electrode.^[39]^ To obtain the QC derivatives, we irradiated the NBD isomers in MeCN by UV light (*λ*_max_=310 nm) (for details see the Experimental Section and the Supporting Information).

The DPVs for NBD1 and QC1 (‐0.8 to 1.2 V_fc_, step size 2 mV, sample period 0.5 s, pulse height 20 mV, pulse time 0.25 s) are depicted in Figure 3a, and the corresponding CVs are shown in Figure S1a of the Supporting Information. In the DPV of NBD1, we observe no specific features below 1.0 V_fc_. Above 1.0 V_fc_, there is an increasing current signal. In sharp contrast, the DPV of QC1 shows an additional peak at 0.55 V_fc_ with an onset at 0.3 V_fc_. The integrated total charge in this feature is 0.018 C. Above 1.0 V_fc_, the increase in current is very similar to the behavior observed for NBD1.

We attribute the increase of current at ≥1.0 V_fc_ to the oxidation of NBD1.^[45]^ For the additional peak at 0.55 V_fc_ in the DPV of QC1, we may invoke two possible explanations. First, the peak may arise from reversible formation of the QC1^+^⋅ radical, which then initiates the back‐conversion. Alternatively, the effect peak may originate from formation of QC1^+^⋅ followed by irreversible oxidative decomposition.^[46]^ We will return to this point later, after discussing the spectroscopic data.

In the next step, we consider the DPVs (see Figure 3b) and CVs (see Figure S1b in the Supporting Information) of the NBD2/QC2 couple. The voltammogram of NBD2 shows one dominating peak at 1.0 V_fc_ with an onset at 0.8 V_fc_ (total charge of 0.021 C). The same feature is observed for QC2. In analogy to the NBD1/QC1 system, we assign this peak to the oxidative decomposition of NBD2. In addition, we observe a very weak feature at 0.45 V_fc_ corresponding to a total charge of 0.002 C, which appears in the DPV of QC2 only. In comparison to the peak observed at low potential for the QC1, this feature is weaker by approximately one order of magnitude. Note that we observed a similar behavior for other NBD derivatives with methoxy groups in previous studies.[^38^, ^39^]

**Figure 4:**
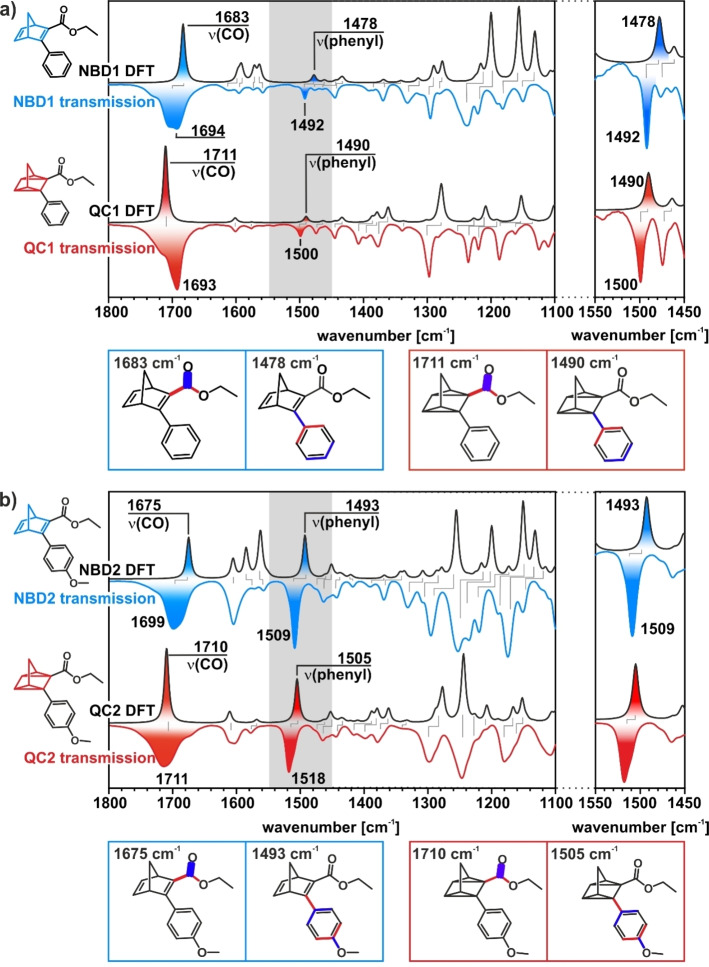
(a) Transmission IR spectra of NBD1 and QC1 and the corresponding spectra calculated by DFT. (b) Transmission IR spectra of NBD2 and QC2 and the corresponding spectra calculated by DFT. In the insets, selected carbonyl and phenyl stretch vibrations are visualized.

To identify the origin of the different features in the DPVs, we performed in‐situ infrared (IR) spectroscopy. First, we analyzed the IR spectra of the two NBD and QC derivatives by comparing transmission IR spectra and simulated IR spectra from DFT. Both the experimental and the simulated spectra are shown in Figure 4a,b for NBD1/QC1 and NBD2/QC2, respectively. A detailed assignment of the bands is provided in the Supporting Information. In the following analysis, we focus on two characteristic bands, which allow us to identify the two photo‐isomers in the NBD1/QC1 and NBD2/QC2 systems by in‐situ IR spectroscopy. These are the carbonyl band around 1700 cm^−1^ and the *ν*(phenyl) band around 1500 cm^−1^ (see visualization of the vibrational modes below the spectra in Figure 4).^[47]^ The carbonyl bands of NBD and QC overlap partially, which results in a characteristic change of the band shape (see the Supporting Information for details). Most importantly, the *ν*(phenyl) band shows a characteristic shift upon conversion from NBD to QC of almost 10 cm^−1^. As a result, this band is well suitable as a spectroscopic marker and allows us to obtain quantitative information on the concentration of the species (see the Supporting Information for details).

**Figure 5:**
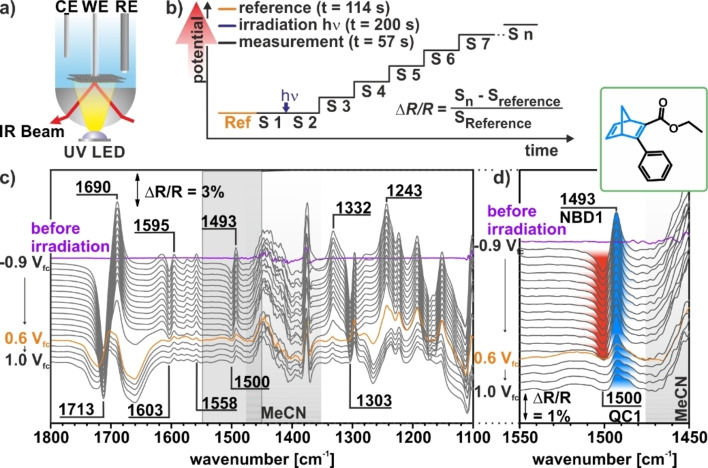
Electrochemically triggered back‐conversion of NBD1. (a) Schematic representation of the PEC‐IRRAS setup. (b) Experimental procedure. (c) IRRAS data of the photochemical conversion and electrochemical back‐conversion in the NBD1/QC1 system on a HOPG electrode. (d) *ν*(CC) region as a function of the electrode potential. The reference spectra were taken at −0.9 V_fc_. CE (counter electrode), WE (working electrode), RE (reference electrode), Ref (reference spectrum), S_*n*_ (spectrum number *n*).

In order to follow the electrochemically triggered back‐conversion of the NBD1/QC1 and NBD2/QC2 systems in situ, we performed potential step photoelectrochemical infrared reflection absorption spectroscopy (PEC‐IRRAS) experiments. The setup and the procedure are schematically depicted in Figures 5a,b. All data were measured in thin‐layer configuration with a liquid film of a few μm between the working electrode (highly oriented pyrolytic graphite, HOPG) and the UV/IR window (see Figure 5a). Note that in this geometry mass transport in the thin layer is decoupled from the bulk solution.^[48]^ We recorded a background spectrum and a first IR spectrum before irradiation at −0.9 V_fc_. Subsequently, the thin layer was irradiated (310 nm, 30 mW, 200 s) and another IR spectrum was recorded (see the Supporting Information for details). The potential was increased stepwise (0.1 V) to 1.0 V_fc_ and one IR spectrum was recorded at each potential step. The corresponding IR spectra of the NBD1/QC1 system are depicted in Figure 5c. All presented spectra are difference spectra referred to the spectrum recorded immediately before irradiation. As a result, negative (pointing downwards) and positive (pointing upwards) bands indicate formed and consumed species, respectively.

After irradiation, we observe positive bands at 1690 cm^−1^ [*ν*(CO)], 1595 cm^−1^ [*ν*(C=C)_phenyl,NBD_], 1558 cm^−1^ [*ν*(C=C)_phenyl,NBD_], 1493 cm^−1^ [*δ*(CH)_phenyl_], 1332 cm^−1^ [*δ*(CH)_phenyl_], and 1243 cm^−1^ [*δ*(CH)_NBD_], which we assign to NBD1. Further, we observe negative bands at 1712 cm^−1^ [*ν*(CO)], 1603 cm^−1^ [*ν*(C=C)_phenyl_], 1500 cm^−1^ [*δ*(CH)_phenyl_], and 1303 cm^−1^ [*ν*(CC)_QC,ester_], which we assign to the formed QC1. We attribute the band at 1450 cm^−1^ to the solvent MeCN.^[49]^ The results indicate photochemical conversion of NBD1 to QC1. When increasing the potential from −0.9 to 0.6 V_fc_, the bands of QC1 disappear, while a positive contribution from NBD1 remains (see, e. g., the spectroscopic marker band at 1493 cm^−1^, Figure 5d). We conclude that QC1 is back‐converted to NBD1 partially, while a fraction of QC1 decomposes.

**Figure 6:**
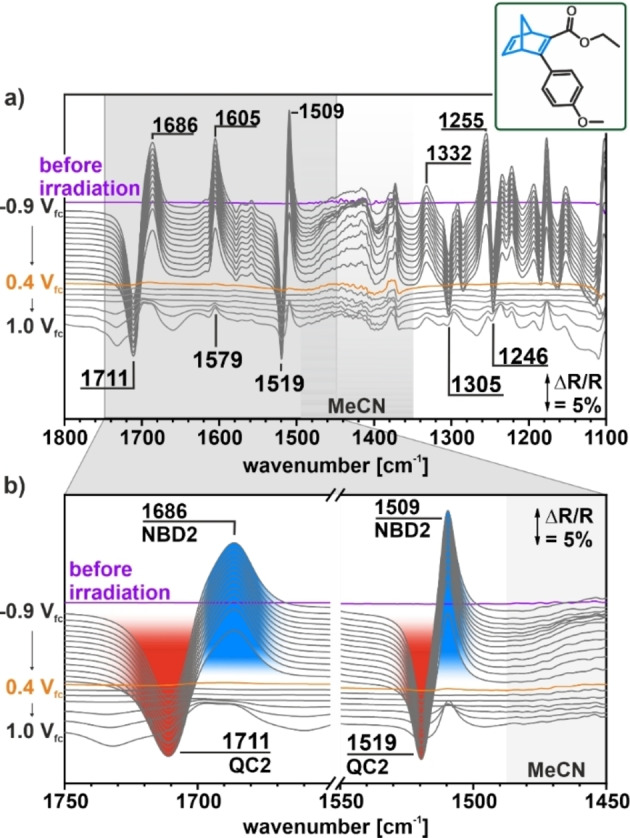
Electrochemically triggered back‐conversion of NBD2. (a) IRRAS data of the photochemical conversion and electrochemical back‐conversion in the NBD2/QC2 system on a HOPG electrode. (b) *ν*(CO) and *ν*(CC) regions as a function of the electrode potential. The reference spectra were taken at −0.9 V_fc_.

In Figure 6, we show the spectra for the equivalent experiment with the NBD2/QC2 system. After irradiation, positive bands at 1686 cm^−1^ [*ν*(CO)], 1605 cm^−1^ [*ν*(C=C)_phenyl,NBD_], 1509 cm^−1^ [*ν*(C=C)_phenyl,NBD_], 1332 cm^−1^ [*δ*(CH)_phenyl_], 1255 cm^−1^ [*δ*(CH)_NBD_] and negative bands at 1711 cm^−1^ [*ν*(CO)], 1579 cm^−1^ [*ν*(C=C, CO)_phenyl_], 1519 cm^−1^ [*δ*(CH)_phenyl_], 1305 cm^−1^ [*δ*(CH)_QC_], and 1255 cm^−1^ [*ν*(COC)_methoxy_] indicate the photochemical conversion from NBD2 to QC2. In sharp contrast to the NBD1/QC1 system, however, all bands vanish completely when the potential is ramped to 0.4 V_fc_. The disappearance of all bands shows that QC2 is quantitatively back‐converted to NBD2 without any indication of decomposition. Based on the band shape and intensities, we estimate that the selectivity of the back conversion is at least 99.3 %. At potentials ≥0.8 V_fc_, positive bands appear, which can be assigned to NBD. This observation indicates oxidative decomposition of the back‐converted NBD in this potential region.

**Figure 7:**
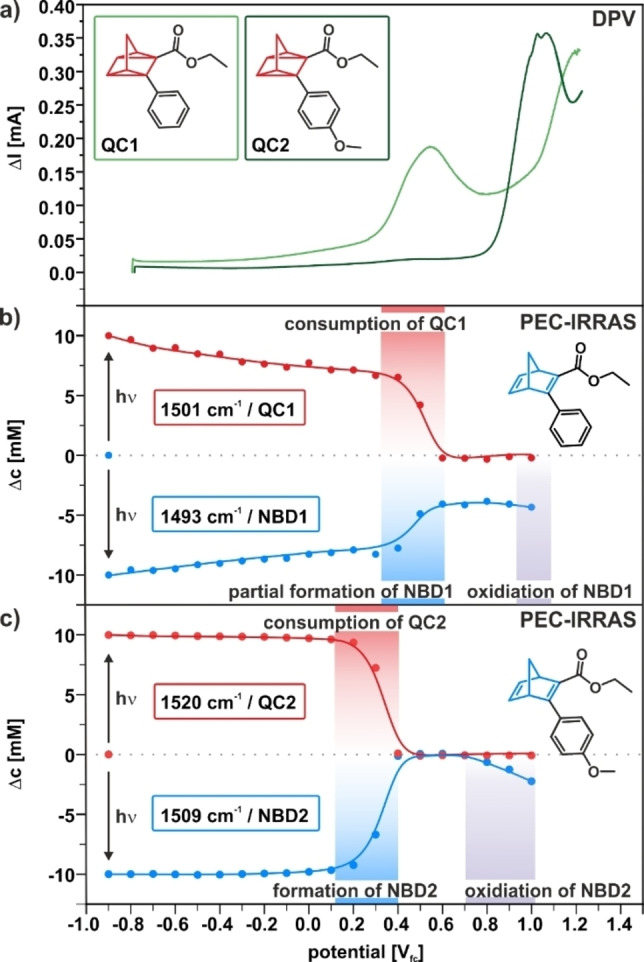
Comparison of the IRRAS data with the electrochemical characterization. (a) Comparison of the differential pulse voltammograms of QC1 and QC2. (b) Change of concentrations of NBD1 and QC1 during the experiment depicted in Figure 5. (c) Change of concentrations of NBD2 and QC2 during the experiment depicted in Figure 6; the concentrations were derived from the band intensities of the *ν*(CC)_phenyl_ bands.

Based on the intensities of the spectroscopic marker bands [*ν*(CC)_phenyl_, ≈1500 cm^−1^], we calculated the concentrations of the NBD and QC species as a function of the potential (see the Supporting Information for details). In Figure 7, we compare the voltammograms (Figure 7a) of the two QC derivatives to the changes in concentration as determined from the PEC‐IRRA spectra of NBD1/QC1 (Figure 7b) and NBD2/QC2 (Figure 7c). For NBD1/QC1, we observe that there is quantitative photochemical conversion, with only partial back‐conversion in the potential range between −0.9 and 0.3 V_fc_. Note that QC1 is a rather stable molecule with a half‐life *t*_1/2_ of 450 days (in tetrachloroethane at 25 °C).^[19]^ Furthermore, we showed that HOPG is catalytically inactive for the back‐reaction.^[39]^ Consequently, we assign the slow back‐conversion at low potential to solvent effects,^[16]^ triggering by the supporting electrolyte,^[50]^ or by reactive species formed during the photochemical treatment. At potentials ≥0.5 V_fc_, we observe that the concentration of QC1 (red) decreases rapidly. Simultaneously, the concentration of NBD1 (blue) increases, but levels off at around −4 mm with respect to the starting concentration. This observation indicates that approximately 60 % of QC is electrochemically back‐converted, while 40 % decomposes. The high degree of decomposition leads to a low cyclability limiting the applicability of the photoswitch for electrochemically triggered MOST devices.

For the NBD2/QC2 system, we find a very different behavior (see Figure 7c). After photochemical conversion, QC2 is perfectly stable in a potential window between −0.9 and 0.2 V_fc_ on the timescale of the experiment. Starting from 0.3 V_fc_, we observe rapid back‐conversion to NBD2. At 0.4 V_fc_, the back‐conversion reaction is completed without any indication for the formation of side products. Note that the onset potential is 0.1 V lower than for QC1. This is in perfect agreement with our theoretical calculations (see Figure 2). In the potential range from 0.4 to 0.7 V_fc_, NBD2 is stable, while above 0.7 V_fc_ we observe consumption of NBD2 due to oxidative decomposition. Our results demonstrate that there is a potential window between 0.4 and 0.7 V_fc_ in which the electrochemically triggered back‐conversion occurs with high selectively (≥99.3 %). In this region, the concentration of side products was below the detection limit of our experiment. This high selectivity leads to good cyclability with around 30 % decomposition after 100 cycles. This value is similar to the NBD/QC derivative, which reached highest cyclability so far using the electrochemically triggered energy release.^[39]^ For a detailed description of the cyclability experiment, we refer to the Supporting Information.

Importantly, we note, that for QC1 there is a strong voltammetric peak (0.55 V_fc_) associated with the loss of this compound, while the corresponding peak (0.45 V_fc_) is very weak for QC2. We assign the two peaks to the formation of the radical cations QC1^+^⋅ and QC2^+^⋅, respectively. We conclude that the voltammetric peak is primarily associated with the irreversible oxidative decomposition of the QC and not with the electrochemically triggered back‐conversion to NBD. The latter process is a chain reaction and only requires charge transfer to start the reaction. Our observations show that already small concentrations of the QC^+^⋅ radical are sufficient to initiate the energy release. We assume that the voltammetric peak for QC1 (0.018 C) corresponds to oxidation of 40 % of QC1 via a one‐electron process and we consider the peak intensity leading to quantitative conversion of QC2 (0.002 C) at the electrode surface (diffusion limitation). Based on these assumptions, we make the very rough estimate that approximately 20 QC2 molecules are back‐converted per QC2^+^⋅ radical formed (for details and assumptions made see the Supporting Information). This implies that the amount of charge transfer required to trigger the energy release is small (see the Supporting Information for details) and, therefore, the electrochemically driven release process hardly affects the total energy efficiency of MOST storage system.

## Conclusion

In this work, we studied the influence of specific substituents on the electrochemically triggered energy release from molecular solar thermal (MOST) systems based on the norbornadiene/quadricyclane (NBD/QC) couple. Specifically, we investigated the derivatives phenyl‐ethyl ester‐norbornadiene/quadricyclane (NBD1/QC1) and *p*‐methoxyphenyl‐ethyl ester‐norbornadiene/quadricyclane (NBD2/QC2), which differ by one methoxy group in the push‐pull system. In our study, we combined in‐situ photoelectrochemical infrared spectroscopy, electrochemical methods (differential pulse voltammetry and cyclic voltammetry), and modelling (density functional theory). From our results, we conclude:

1. Electrochemical triggering: For both QC derivatives, it is feasible to trigger the energy release electrochemically. The reactions follow an oxidative reaction channel with a QC^+^⋅ radical intermediate which initiates a chain reaction. The presence of the additional methoxy group in QC2 lowers the onset potential of the back‐conversion by around 0.1 V.
2. Selectivity of the electrochemically triggered back‐conversion: The selectivity of the electrochemically triggered energy release reaction differs drastically for the two compounds. QC1 is back‐converted electrochemically to NBD1 with a selectivity of only 60 %. By introducing the additional methoxy group in NBD2, the selectivity increases significantly. The in‐situ IR experiment shows quantitative conversion and no indication of side reactions (selectivity ≥99.3 %). The electrochemically triggered back‐conversion occurs in a potential window from 0.4 to 0.7 V_fc_.
3. Electrochemical response: QC1 shows an additional electrochemical response in the voltammogram at 0.6 V_fc_. The corresponding feature for QC2 appears at 0.45 V_fc_; however, it is much weaker for this compound (by approximately a factor of 10). We assign these signals to the electrochemical oxidation of QC1/QC2. Whereas the formation of QC1^+^⋅ radical cations leads in part to irreversible oxidation of QC1, the formation of QC2^+^⋅ triggers the back‐conversion to NBD2 without major side reactions. As the latter process is a radical chain reaction, only a very small total charge is required to back‐convert the QC2.
4. Impact on MOSTs: Our results indicate that already low concentrations of QC^+^⋅ radicals are sufficient to trigger the back‐conversion quantitatively. A rough estimate suggests that approximately 20 NBD2 units are back‐converted per QC2^+^⋅ radical cation formed. This charge‐efficient release mechanism has substantial implications for potential applications. It means that the energy input to trigger the energy release electrochemically is very low and, for a properly designed system, it will hardly affect the total energy efficiency of MOST storage system.

Our results demonstrate that proper molecular design is essential for the electrochemical stability of MOST systems. A better understanding of underlying structure/stability correlations will be a key for the design of electrochemically controlled MOST technologies.

## Experimental Section

### Cleaning

All Teflon and glass ware as well as noble metal wires were stored in sulfuric acid (Merck, Emsure, 98 %) with NOCHROMIX® (Sigma Aldrich) for at least one night. Before each use, the equipment was rinsed with ultra‐pure water (MilliQ Synergy UV, 18.2 MΩ cm at 25 °C, TOC <5 ppb) for 5 times and boiled 3 times in ultra‐pure water for 30 min. Afterwards it was dried under vacuum overnight.

### Electrochemical investigation

PEC‐IRRAS experiments were performed using a HOPG crystal (MikroMasch, ZYA, 0.4° mosaic spread) as working electrode (WE), which was cleaned prior each measurement by cleavage using scotch tape. For CVs and DPVs we used a graphite rod. We used a solution of 10 mm NBD1 or NBD2 in 0.1 m Bu_4_NClO_4_ (Sigma Aldrich, ≥99.0 %) as supporting electrolyte in MeCN (Sigma Aldrich, 99.999 % trace metals basis). The potential was applied using a commercial potentiostat (Gamry, Reference [60]) with a three‐electrode setup. We used a graphite rod as counter electrode (CE) and an Ag/Ag^+^ (0.01 m AgNO_3_ with 0.1 m Bu_4_NClO_4_ in MeCN) electrode as reference electrode (RE). The RE was calibrated before and after each measurement day versus the redox potential of ferrocene (Alfa Aesar, 99.5 %), determined by CV. Note that in this work, we referred all potentials to the redox potential of the ferrocene couple (V_fc_).

### CV and DPV

We performed all CVs with a scan rate of 50 mV s^−1^. The DPVs were performed with a step size of 2 mV, a sample period of 0.5 s, a pulse size of 20 mV, and a pulse time of 0.25 s.

### PEC‐IRRAS

To measure PEC‐IRRAS, we used a vacuum‐based Fourier‐transform infrared (FTIR) spectrometer (Bruker, Vertex 80v) with evacuated optics and a liquid‐nitrogen‐cooled mercury cadmium telluride (MCT) detector. The photochemical conversion was performed with an UV LED (Seoul Viosys, CUD1AF4D, 310 nm, 30 mW). This was located below an IR and UV transparent CaF_2_ (Korth, *d*=25 mm) hemisphere. The IR detector was protected from UV light with a KRS‐5 filter. All in‐situ measurements were measured in reflection mode in thin layer configuration. Potential dependent spectra were recorded with a resolution of 2 cm^−1^, a scanner velocity of 40 kHz, 128 scans per spectrum (background 256 scans per spectrum) and an acquisition time of 57 s (background 114 s). Time resolved spectra were recorded with a resolution of 8 cm^−1^, a scanner velocity of 240 kHz and an acquisition time of 21 ms per scan. For all measurements, we used non‐polarized light. We recorded transmission spectra using KBr (≥99 %, FTIR‐grade, Sigma Aldrich) pellets. For the transmission spectra of the QC isomers we irradiated the KBr pellet (310 nm, 30 mW) until the resulting IR spectra did not change (≈1 h).

### DFT calculations

DFT calculations were performed for molecules in the gas phase using the Turbomole Software package version 7.2.^[51]^ The basis set, def2 TZVP,^[52]^ was used with the exchange‐correlation functional PBE^[43]^ and B3LYP.^[44]^ To accelerate the calculations the RI‐J approximation^[53]^ was applied. The D3 correction scheme^[54]^ was included to account for long range dispersion interactions. To include solvent effects the COSMO solvation model^[55]^ was applied with a dielectric constant of 37.5 for the used solvent acetonitrile. The vibrational spectra were calculated using harmonic frequency calculations with analytical gradients. To visualize the data QVibeplot^[47]^ was used.

### Synthesis of NBDs

**Figure 8:**
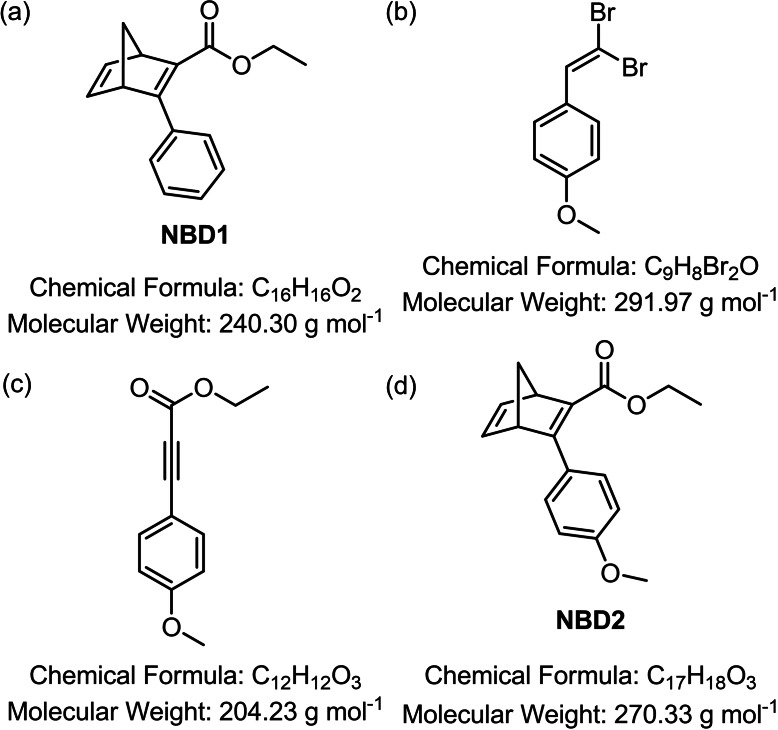
Structures and parameters of the synthesized products. (a) NBD1, (b) 1‐(2,2‐dibromovinyl)‐4‐methoxybenzene, (c) ethyl 3‐(4‐methoxyphenyl)propiolate, (d) NBD2.

**Ethyl 3‐(4‐phenyl)bicyclo[2.2.1]hepta‐2,5‐diene‐2‐carboxylate (phenyl‐ethyl ester‐NBD, NBD1)**: Freshly cracked cyclopentadiene (1.35 mL, 16.3 mmol, 1.3 equiv.), ethyl phenylpropiolate (2.07 mL, 12.5 mmol, 1.0 equiv.), and toluene (3 mL) were combined in a microwave vial equipped with a stirring bar. The reaction vessel was sealed, and the solution degassed for 10 min using an argon stream. The mixture was heated to 180 °C for 4 h using a microwave reactor. The solvent was removed under reduced pressure. Purification was achieved via fractionated distillation over a Vigreux column (bp=123 °C at 4.6×10^−2^ mbar) yielding the desired product (Figure 8a) as colorless oil which solidified while storing in the freezer (‐20 °C). Yield: 1.35 g, 5.62 mmol, 45 %. ^1^H NMR (400 MHz, CDCl_3_, 25 °C): *δ*_H_=7.54–7.50 (m, 2H), 7.39–7.28 (m, 3H), 7.01–6.98 (m, 1H), 6.94–6.91 (m, 1H), 4.14 (qd, *J*_1_=7.1 Hz, *J*_2_=0.8 Hz, 2H), 4.08–4.05 (m, 1H), 3.87–3.84 (m, 1H), 2.26 (dt, *J*_1_=6.6 Hz, *J*_2_=1.6 Hz, 1H), 2.06 (dt, *J*_1_=6.6 Hz, *J*_2_=1.6 Hz, 1H), 1.22 ppm (t, *J*_1_=7.1 Hz, 3H).

**1‐(2,2‐Dibromovinyl)‐4‐methoxybenzene**: To a stirred 0 °C cold solution of CBr_4_ (5.18 g, 15.6 mmol, 1.3 equiv.) in CH_2_Cl_2_ (20 mL), PPh_3_ (8.19 g, 31.2 mmol, 2.6 equiv.) was added portion‐wise. The mixture was stirred for 1 h at 0 °C before 4‐methoxybenzaldehyde (1.46 mL, 12.0 mmol, 1.0 equiv.) and triethylamine (1.67 mL, 12.0 mmol, 1.0 equiv.) were added. The stirring was continued for further 2 h at 0 °C. Hexanes (200 mL) were added to the reaction resulting in the formation of an orange precipitate. The mixture was stored in the freezer (‐20 °C) over night to maintain complete precipitation and phase separation. The liquid phase was decanted off and the solid residue extracted with a mixture of 2 : 1 hexanes/CH_2_Cl_2_ (3×100 mL). The before separated liquid phase and the extracts were combined and filtered through a plug of silica gel eluted with hexanes/CH_2_Cl_2_ (2 : 1). The filtrate was concentrated under reduced pressure to yield the target compound (Figure 8b) as pale‐yellow solid. Yield: 2.66 g, 9.09 mmol, 76 %. ^1^H NMR (400 MHz, CDCl_3_, 25 °C): *δ*_H_=7.53–7.49 (m, 2H), 7.41 (s, 1H), 6.91–6.87 (m, 2H), 3.82 ppm (s, 3H).

**Ethyl 3‐(4‐methoxyphenyl)propiolate**: In a flame‐dried apparatus purged with N_2_, 1‐(2,2‐dibromovinyl)‐4‐methoxybenzene (1.79 g, 6.15 mmol, 1.0 equiv.) was dissolved in dry THF (25 mL). The solution was cooled to −78 °C before *n*‐BuLi (2.5 m solution in hexanes, 5.40 mL, 13.5 mmol, 2.2 equiv.) was added dropwise over a duration of 30 min. After stirring for 1 h at this temperature, ethyl chloroformate (0.882 mL, 9.23 mmol, 1.5 equiv.) was added at once at −78 °C. The mixture was allowed to reach room temperature and was stirred for an additional hour at this temperature. The reaction was quenched through addition of H_2_O (25 mL). The two phases were separated, and the aqueous phase extracted with ethyl acetate (3×30 mL). The combined organic phases were dried over MgSO_4_, filtered, and the solvent removed under reduced pressure. Purification was achieved via flash column chromatography (SiO_2_, hexanes/CH_2_Cl_2_, 3 : 1→1 : 1, *v*/*v*) yielding the desired compound (Figure 8c) as yellow oil, which solidified while storing in the freezer (−20 °C). Yield: 1.01 g, 4.95 mmol, 80 %. ^1^H NMR (400 MHz, CDCl_3_, 25 °C): *δ*_H_=7.53–7.49 (m, 2H), 6.88–6.83 (m, 2H), 4.26 (q, *J*_1_=7.1 Hz, 2H), 3.80 (s, 3H), 1.33 ppm (t, *J*_1_=7.1 Hz, 3H).

**Ethyl 3‐(4‐methoxyphenyl)bicyclo[2.2.1]hepta‐2,5‐diene‐2‐carboxylate (*p–*methoxyphenyl‐ethyl ester‐NBD, NBD2)**: Freshly cracked cyclopentadiene (0.532 mL, 6.44 mmol, 1.3 equiv.), ethyl 3‐(4‐methoxyphenyl)propiolate (1.01 g, 4.95 mmol, 1.0 equiv.), and toluene (6 mL) were combined in a microwave vial equipped with a stirring bar. The reaction vessel was sealed, and the solution degassed with N_2_ for 15 min. The mixture was heated to 180 °C for 5 h using a microwave reactor. After complete reaction time, the solvent was removed under reduced pressure. Purification was achieved via twofold flash column chromatography (SiO_2_, 1. hexanes/CH_2_Cl_2_, 3 : 2→1 : 3, *v*/*v*; 2. hexanes/CH_2_Cl_2_, 1 : 3, *v*/*v*) yielding the desired product (Figure 8d) as yellow oil. Yield: 739 mg, 2.73 mmol, 55 %. ^1^H NMR (400 MHz, CDCl_3_, 25 °C): *δ*_H_=7.59–7.55 (m, 2H), 6.98–6.95 (m, 1H), 6.91–6.87 (m, 3H), 4.15 (qd, *J*_1_=6.8 Hz, *J*_2_=2.5 Hz, 2H), 4.06–4.03 (m, 1H), 3.86–3.84 (m, 1H), 3.83 (s, 1H), 2.21 (dt, *J*_1_=6.6 Hz, *J*_2_=1.6 Hz, 1H), 2.03 (dt, *J*_1_=6.6 Hz, *J*_2_=1.6 Hz, 1H), 1.25 ppm. (t, *J*_1_=7.1 Hz, 3H). For further details we refer to the literature.^[19]^

## Conflict of interest

The authors declare no conflict of interest.

1

## Supporting information

As a service to our authors and readers, this journal provides supporting information supplied by the authors. Such materials are peer reviewed and may be re‐organized for online delivery, but are not copy‐edited or typeset. Technical support issues arising from supporting information (other than missing files) should be addressed to the authors.

Supporting Information

## Data Availability

The data that support the findings of this study are available from the corresponding author upon reasonable request.

## References

[1] Z. Wang , P. Erhart , T. Li , Z.-Y. Zhang , D. Sampedro , Z. Hu , H. A. Wegner , O. Brummel , J. Libuda , M. B. Nielsen , K. Moth-Poulsen , Joule 2021, 5, 3116–3136.

[2] M. J. Kuisma , A. M. Lundin , K. Moth-Poulsen , P. Hyldgaard , P. Erhart , J. Phys. Chem. C 2016, 120, 3635–3645. 10.1021/acs.jpcc.5b11489 PMC4780837 26966476

[3] T. J. Kucharski , N. Ferralis , A. M. Kolpak , J. O. Zheng , D. G. Nocera , J. C. Grossman , Nat. Chem. 2014, 6, 441–447. 24755597 10.1038/nchem.1918

[4] A. Lennartson , A. Roffey , K. Moth-Poulsen , Tetrahedron Lett. 2015, 56, 1457–1465.

[5] H.-D Scharf , J. Fleischhauer , H. Leismann , I. Ressler , W.-G. Schleker , R. Weitz , Angew. Chem. Int. Ed. Engl. 1979, 18, 652–662.

[6] T. J. Kucharski , Y. Tian , S. Akbulatov , R. Boulatov , Energy Environ. Sci. 2011, 4, 4449–4472.

[7] A. Goulet-Hanssens , M. Utecht , D. Mutruc , E. Titov , J. Schwarz , L. Grubert , D. Bléger , P. Saalfrank , S. Hecht , J. Am. Chem. Soc. 2017, 139, 335–341. 27997152 10.1021/jacs.6b10822

[8] Z. F. Liu , K. Hashimoto , A. Fujishima , Nature 1990, 347, 658–659.

[9] A. H. Heindl , H. A. Wegner , Chem. Eur. J. 2020, 26, 13730–13737. 32330338 10.1002/chem.202001148 PMC7702042

[10] J. H. Griwatz , A. Kunz , H. A. Wegner , Beilstein J. Org. Chem. 2022, 18, 781–787. 35859625 10.3762/bjoc.18.78 PMC9263553

[11] S. L. Broman , M. B. Nielsen , Phys. Chem. Chem. Phys. 2014, 16, 21172–21182. 25175333 10.1039/c4cp02442g

[12] K. Edel , X. Yang , J. S. A. A. Ishibashi , A. N. Lamm , C. Maichle-Mössmer , Z. X. Giustra , S.-Y. Y. Liu , H. F. Bettinger , Angew. Chem. Int. Ed. 2018, 57, 5296–5300; 10.1002/anie.201712683 PMC6214188 29457683

[13] J. J. Zou , Y. Liu , L. Pan , L. Wang , X. Zhang , Appl. Catal. B 2010, 95, 439–445.

[14] A. M. Kolpak , J. C. Grossman , J. Chem. Phys. 2013, 138, 34303. 10.1063/1.4773306 23343272

[15] C. Schuschke , C. Hohner , M. Jevric , A. Ugleholdt Petersen , Z. Wang , M. Schwarz , M. Kettner , F. Waidhas , L. Fromm , C. J. Sumby , A. Görling , O. Brummel , K. Moth-Poulsen , J. Libuda , Nat. Commun. 2019, 10, 2384. 31160590 10.1038/s41467-019-10263-4 PMC6546758

[16] M. Quant , A. Hamrin , A. Lennartson , P. Erhart , K. Moth-Poulsen , J. Phys. Chem. C 2019, 123, 7081–7087.

[17] V. A. Bren’ , A. D. Dubonosov , V. I. Minkin , V. A. Chernoivanov , Russ. Chem. Rev. 1991, 60, 451–469.

[18] P. A. Grutsch , C. Kutal , J. Am. Chem. Soc. 1986, 108, 3108–3110.

[19] P. Lorenz , T. Luchs , A. Hirsch , Chem. Eur. J. 2021, 27, 4993–5002. 33449419 10.1002/chem.202005427 PMC7986914

[20] M. Quant , A. Lennartson , A. Dreos , M. Kuisma , P. Erhart , K. Börjesson , K. Moth-Poulsen , Chem. Eur. J. 2016, 22, 13265–13274. 27492997 10.1002/chem.201602530 PMC5096010

[21] M. Jevric , A. U. Petersen , M. Mansø , S. Kumar Singh , Z. Wang , A. Dreos , C. Sumby , M. B. Nielsen , K. Börjesson , P. Erhart , K. Moth-Poulsen , Chem. Eur. J. 2018, 24, 12767–12772. 29978927 10.1002/chem.201802932

[22] V. Gray , A. Lennartson , P. Ratanalert , K. Börjesson , K. Moth-Poulsen , Chem. Commun. 2014, 50, 5330–5332. 10.1039/c3cc47517d 24280803

[23] M. Mansø , A. U. Petersen , Z. Wang , P. Erhart , M. B. Nielsen , K. Moth-Poulsen , Nat. Commun. 2018, 9, 1945. 29769524 10.1038/s41467-018-04230-8 PMC5956078

[24] M. Nucci , A. Núñez , L. M. Frutos , M. Marazzi , Adv. Sustainable Syst. 2022, 6, 202200097.

[25] M. Nucci , M. Marazzi , L. M. Frutos , ACS Sustainable Chem. Eng. 2019, 7, 19496–19504.

[26] Z. Wang , H. Hölzel , K. Moth-Poulsen , Chem. Soc. Rev. 2022, 51, 7313–7326. 35726574 10.1039/d1cs00890k PMC9426646

[27] T. Luchs , P. Lorenz , A. Hirsch , ChemPhotoChem 2020, 4, 52–58.

[28] K. Maruyama , H. Tamiaki , J. Org. Chem. 1986, 51, 602–606.

[29] K. Maruyama , K. Terada , Y. Yamamoto , Chem. Lett. 1981, 10, 839–842.

[30] K. Maruyama , H. Tamiaki , S. Kawabata , J. Chem. Soc. Perkin Trans. 2 1986, 543–549.

[31] Z. Wang , A. Roffey , R. Losantos , A. Lennartson , M. Jevric , A. U. Petersen , M. Quant , A. Dreos , X. Wen , D. Sampedro , K. Börjesson , K. Moth-Poulsen , Energy Environ. Sci. 2019, 12, 187–193.

[32] D. J. Fife , K. W. Morse , W. M. Moore , J. Am. Chem. Soc. 1983, 105, 7404–7407.

[33] R. Eschenbacher , T. Xu , E. Franz , R. Löw , T. Moje , L. Fromm , A. Görling , O. Brummel , R. Herges , J. Libuda , Nano Energy 2022, 95, 107007.

[34] U. Bauer , S. Mohr , T. Döpper , P. Bachmann , F. Späth , F. Düll , M. Schwarz , O. Brummel , L. Fromm , U. Pinkert , A. Görling , A. Hirsch , J. Bachmann , H.-P. Steinrück , J. Libuda , C. Papp , Chemistry 2017, 23, 1613–1622. 27870528 10.1002/chem.201604443

[35] U. Bauer , L. Fromm , C. Weiß , P. Bachmann , F. Späth , F. Düll , J. Steinhauer , W. Hieringer , A. Görling , A. Hirsch , H. P. Steinrück , C. Papp , J. Phys. Chem. C 2019, 123, 7654–7664. 10.1063/1.5095583 31091921

[36] U. Bauer , L. Fromm , C. Weiß , F. Späth , P. Bachmann , F. Düll , J. Steinhauer , S. Matysik , A. Pominov , A. Görling , A. Hirsch , H. P. Steinrück , C. Papp , J. Chem. Phys. 2019, 150, 184706. 31091921 10.1063/1.5095583

[37] O. Brummel , D. Besold , T. Döpper , Y. Wu , S. Bochmann , F. Lazzari , F. Waidhas , U. Bauer , P. Bachmann , C. Papp , H.-P. Steinrück , A. Görling , J. Libuda , J. Bachmann , ChemSusChem 2016, 9, 1424–1432. 27094340 10.1002/cssc.201600127

[38] F. Waidhas , M. Jevric , L. Fromm , M. Bertram , A. Görling , K. Moth-Poulsen , O. Brummel , J. Libuda , Nano Energy 2019, 63, 103872.

[39] F. Waidhas , M. Jevric , M. Bosch , T. Yang , E. Franz , Z. Liu , J. Bachmann , K. Moth-Poulsen , O. Brummel , J. Libuda , J. Mater. Chem. A 2020, 8, 15658–15664.

[40] K. Yasufuku , K. Takahashi , C. Kutal , Tetrahedron Lett. 1984, 25, 4893–4896.

[41] P. G. Gassman , J. W. Hershberger , J. Org. Chem. 1987, 52, 1337–1339.

[42] O. Brummel , F. Waidhas , U. Bauer , Y. Wu , S. Bochmann , H. P. Steinrück , C. Papp , J. Bachmann , J. Libuda , J. Phys. Chem. Lett. 2017, 8, 2819–2825. 28565910 10.1021/acs.jpclett.7b00995

[43] J. P. Perdew , K. Burke , M. Ernzerhof , Phys. Rev. Lett. 1996, 77, 3865–3868. 10062328 10.1103/PhysRevLett.77.3865

[44] Alex D. Beck , J. Chem. Phys. 1993, 98, 5648–5656.

[45] P. G. Gassman , R. Yamaguchi , G. F. Koser , J. Org. Chem. 1978, 43, 4392–4393.

[46] K. White , D. A. Buttry , J. Electrochem. Soc. 2000, 147, 266.

[47] M. Laurin , J. Chem. Educ. 2013, 90, 944–946.

[48] T. Iwasita , F. C. Nart , J. Electroanal. Chem. 1990, 295, 215–224.

[49] E. L. Pace , L. J. Noe , J. Chem. Phys. 1968, 49, 5317–5325.

[50] E. Franz , A. Kunz , N. Oberhof , A. H. Heindl , M. Bertram , L. L. Fusek , N. Taccardi , P. Wasserscheid , A. Dreuw , H. A. Wegner , O. Brummel , J. Libuda , ChemSusChem 2022, 15, e202200958. 35762102 10.1002/cssc.202200958 PMC9796447

[51] T. G. TURBOMOLE V7.2 2017, a development of University of Karlsruhe and Forschungszentrum Karlsruhe GmbH, 1989–2007, **2007**.

[52] F. Weigend , R. Ahlrichs , Phys. Chem. Chem. Phys. 2005, 7, 3297–3305. 16240044 10.1039/b508541a

[53] F. Weigend , Phys. Chem. Chem. Phys. 2006, 8, 1057–1065. 16633586 10.1039/b515623h

[54] S. Grimme , J. Antony , S. Ehrlich , H. Krieg , J. Chem. Phys. 2010, 132, 154104. 20423165 10.1063/1.3382344

[55] A. Schäfer , A. Klamt , D. Sattel , J. C. W. Lohrenz , F. Eckert , Phys. Chem. Chem. Phys. 2000, 2, 2187–2193.

