# Supplementary material for: Electrocatalytic Energy Release of Norbornadiene‐Based Molecular Solar Thermal Systems: Tuning the Electrochemical Stability by Molecular Design
Source: ChemSusChem. 2022 Nov 11;15(24):e202201483. doi: 10.1002/cssc.202201483 (PMC10099746; doi:10.1002/cssc.202201483)
Supplement: Supplementary file 1 — Supporting Information [file CSSC-15-0-s001.pdf]

ChemSusChem

Supporting Information

Electrocatalytic Energy Release of Norbornadiene-Based Molecular Solar Thermal Systems: Tuning the Electrochemical Stability by Molecular Design

Evanie Franz, Daniel Krappmann, Lukas Fromm, Tobias Luchs, Andreas Görling, Andreas Hirsch, Olaf Brummel,* and Jörg Libuda© 2022 The Authors. ChemSusChem published by Wiley-VCH GmbH. This is an open access article under the terms of the Creative Commons Attribution License, which permits use, distribution and reproduction in any medium, provided the original work is properly cited.

Supporting Information

Electrocatalytic Energy Release of Norbornadiene-Based Molecular Solar Thermal Systems – Tuning the Electrochemical Stability by Molecular Design

Evanie Franz[a], Daniel Krappmann[b], Lukas Fromm[c], Tobias Luchs[b], Andreas Görling[c], Andreas Hirsch[b], Olaf Brummel[a]*, Jörg Libuda[a]

[a] Interface Research and Catalysis, Erlangen Center for Interface Research and Catalysis, Friedrich-Alexander-Universität Erlangen-Nürnberg, Egerlandstraße 3, 91058 Erlangen, Germany.

[b] Chair of Organic Chemistry II, Friedrich-Alexander-Universität Erlangen-Nürnberg, Nikolaus-Fiebiger-Straße 10, 91058 Erlangen, Germany.

[c] Lehrstuhl für Theoretische Chemie, Friedrich-Alexander-Universität Erlangen-Nürnberg, Egerlandstraße 3, 91058 Erlangen, Germany.

*corresponding author: Olaf Brummel olaf.brummel@fau.de

1. **CVs of NBD1/QC1 and NBD2/QC2**

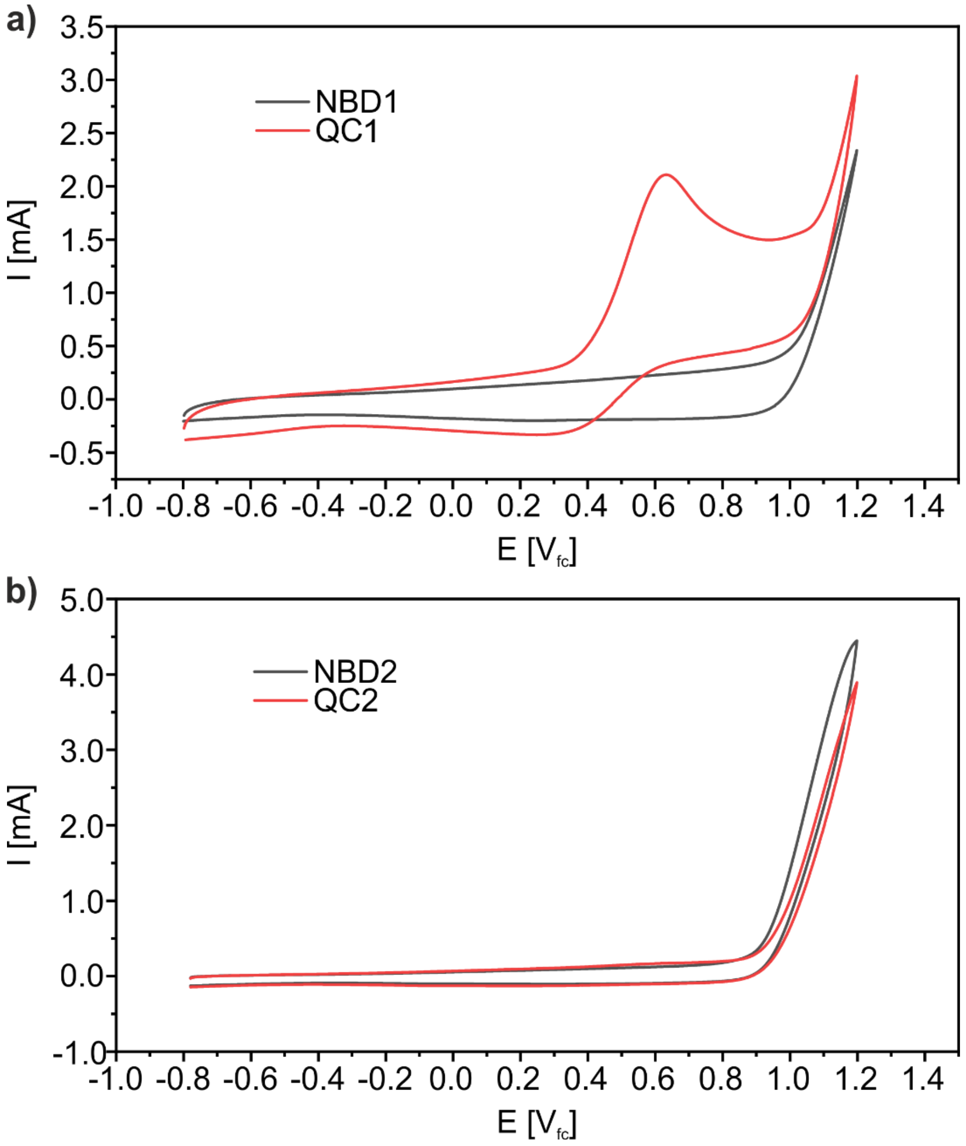

Figure SI1: (a) CVs of NBD1 (black) and QC1 (red); (b) CVs of NBD2 (black) and QC2 (red). All CVs were recorded in a solution of 10 mM compound in 0.1 M TBAP in MeCN with a scan rate of 50 mV/s.

Figure SI1 shows the CVs of NBD1 and NBD2 and their corresponding QC derivates. We used solutions of 10 mM compound in 0.1 M TBAP in MeCN, graphite as WE, CE and Ag/Ag^+ as RE. We cycled the potential between -0.8 V_{fc} and 1.2 V_{fc} with a scan rate of 50 mV/s. To record the CVs of QC1 and QC2, we irradiated NBD1 and NBD2 for 3 hours before the measurement. The resulting CVs of NBD1 and QC1 are depicted in Figure S1a. Similar to the DPV (Figure 3, main text), we observe a decomposition peak in both cases with an onset of 1.0 V_{fc}. In the

CV of QC1 an additional feature arises at an on-set potential of 0.4 V_{fc} and with a maximum at 0.6 V_{fc}.

In Figure S1b, we show the CVs of NBD2 and QC2. The CVs do not show significant differences between each other. Especially the additional peak observable for QC1 compared to NBD1 is very weak and hardly visible in the CV of QC2. The results from the CVs confirm the observations from DPV.

2. Quantification of the solutions used for voltammetry

To make sure that the solutions used for voltammetric studies contain the QC derivate and no side products, we performed ^{1}H-NMR spectroscopy. We irradiated a solution of 10 mM NBD1 in MeCN-d_3 for 2 hours at 310 nm and recorded one NMR spectrum before and after the irradiation (see Figure SI2). For the quantification, we used the quartet signals at δ = 3.94 ppm (NBD1) and δ = 4.09 ppm (QC1). Both signals correspond to two hydrogen atoms and do not overlap with another signal. Based on the integrals, we calculated a ratio of approximately 67$\%_{mol}$ QC1 to 33$\%_{mol}$ NBD1. For NBD2, we performed the same experiment. We used a 10 mM NBD2 in MeCN-d_3 solution and irradiated it for 2 hours at 310 nm. The NMR spectra before and after irradiation are shown in Figure SI3. We determined the ratio between NBD2 and QC2 using the multiplet signals at δ = 7.55 ppm and δ = 7.18 ppm, corresponding to NBD2 and QC2, respectively. We calculated a ratio of approximately 90$\%_{mol}$ QC2 to 10$\%_{mol}$ NBD2. In both cases, we only observe traces of decomposition upon irradiation. For further details on the NMR spectra of both compounds, we refer to literature.[1]

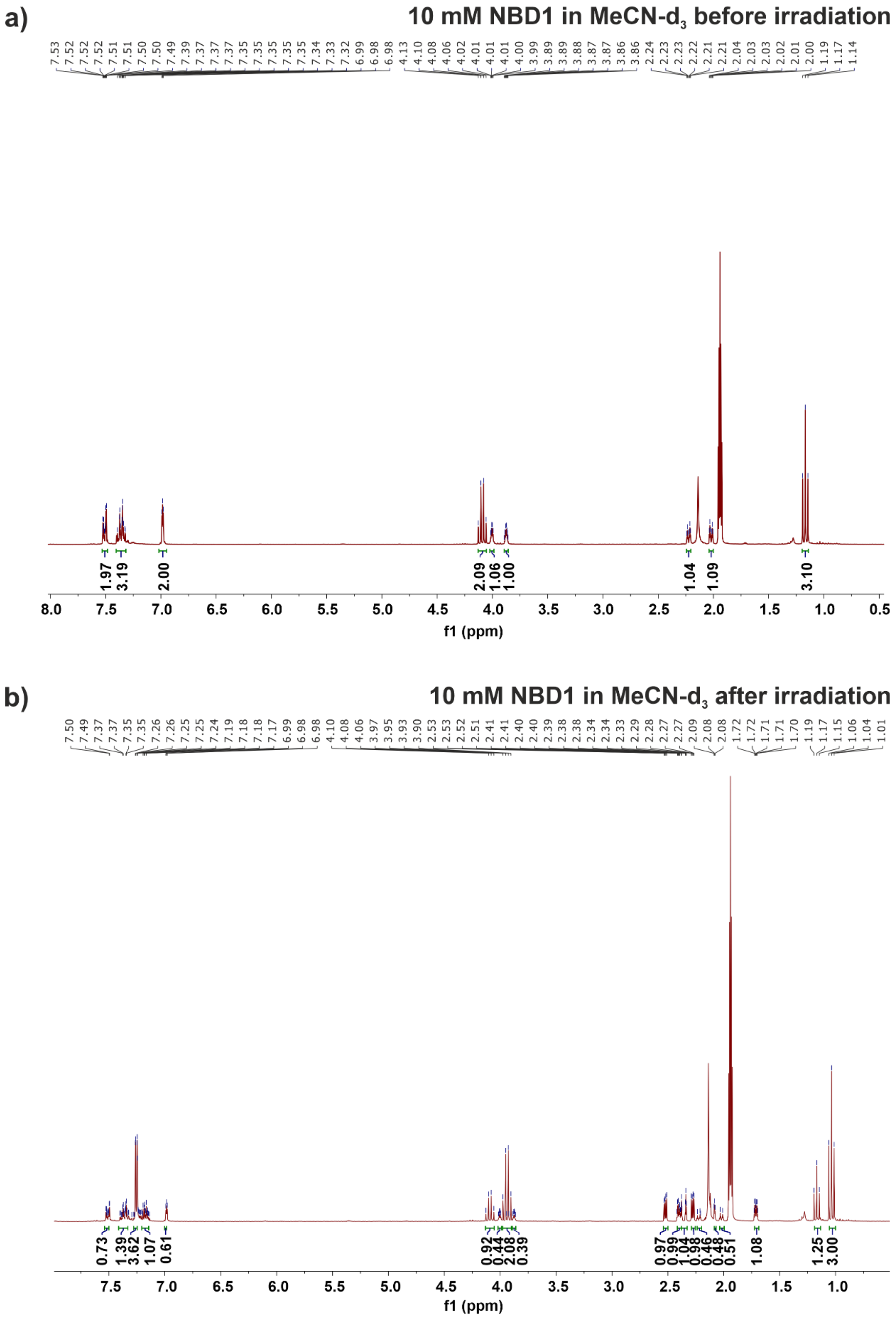

Figure SI2: ^{1}H NMR spectra in MeCN-d_3 of a 10 mM NBD1 solution; a) before irradiation; b) after irradiation at 310 nm (2 hours).

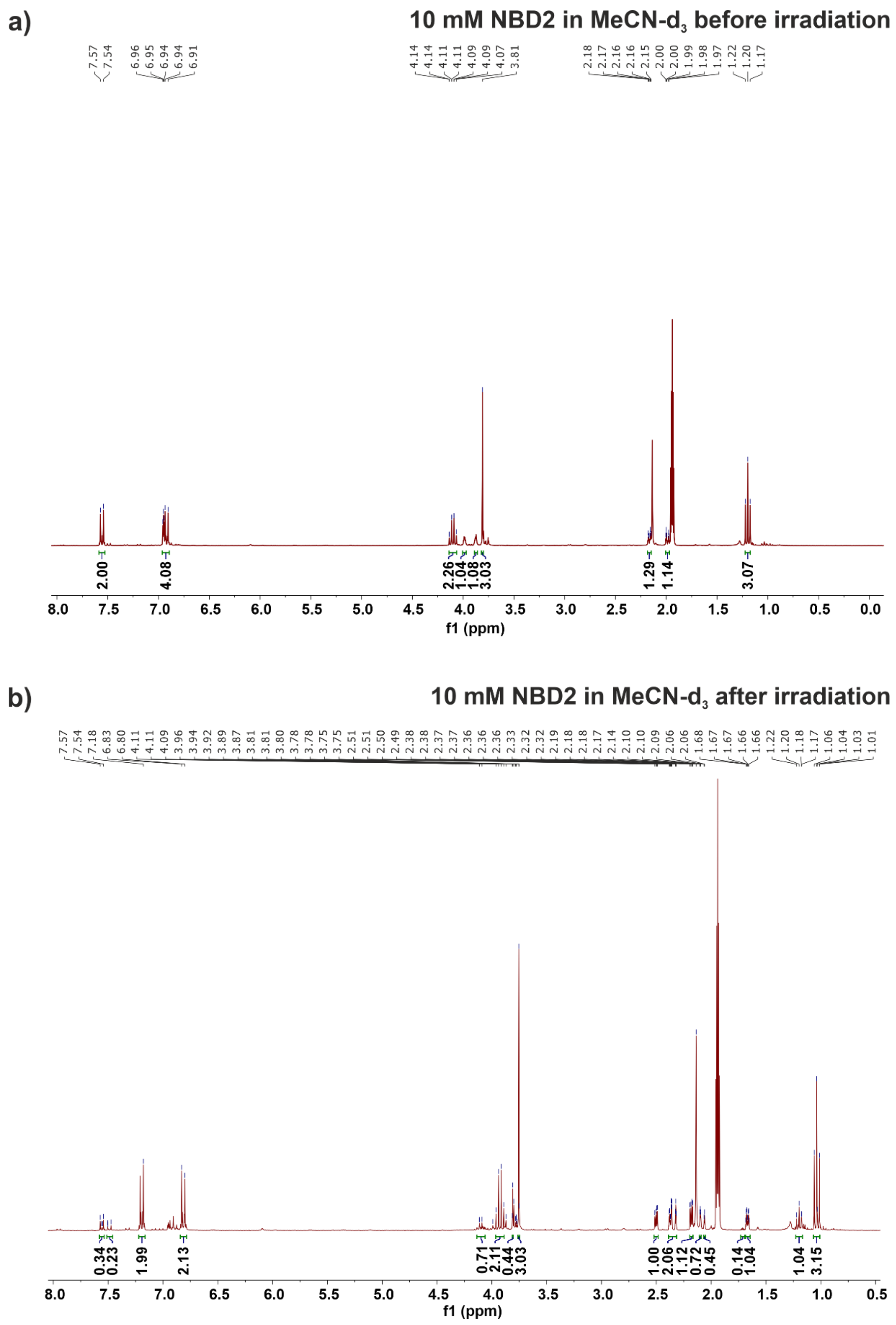

Figure SI3: ^{1}H NMR spectra in MeCN-d_3 of a 10 mM NBD2 solution; a) before irradiation; b) after irradiation at 310 nm (2 hours).

3. **Reference spectra**

The bands of the experimental spectra of NBD1/QC1 (a) and NBD2/QC2 (b) were assigned to the vibrational modes as described in Table SI1 to SI4:

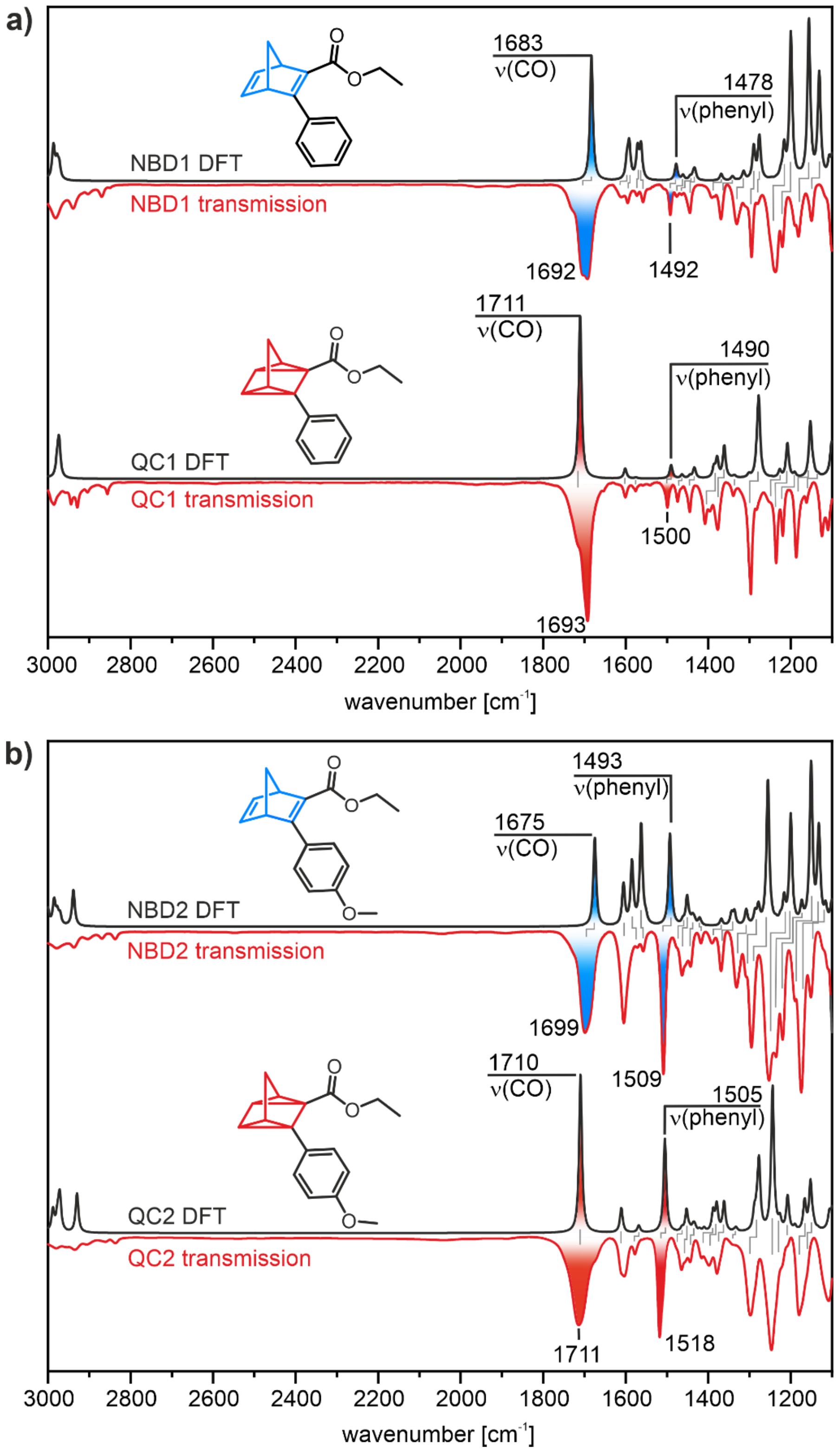

Figure SI4: Transmission and DFT spectra of (a) NBD1 and QC1; (b) NBD2 and QC2.

4. Assignment of the IR-bands

In the following tables (Table SI1 to SI4) we assigned the most intense calculated vibrational modes (in gas phase) to the peaks we observed in the transmission spectra. We visualized the vibrational modes using the program QVibeplot.[2]

Table SI1: Peak assignment for NBD1 based on transmission spectra and DFT calculations.

**Band positions in the transmission spectrum of QC1 [cm^{-1}]**	**Theoretical predicted band positions of QC1 by DFT [cm^{-1}]**	**Vibrational mode**	**2D representations of the vibrational modes**
1150	1131	$\delta(CH)_{NBD}$, $\nu(CC)_{NBD}$	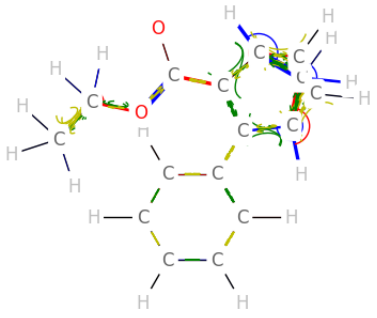
1182	1156	$\delta(CH)_{NBD}$, $\nu(CC)_{NBD}$	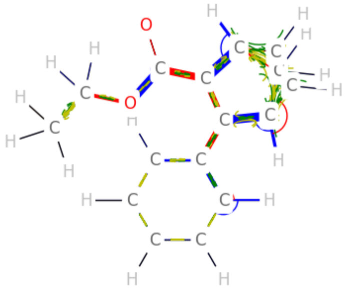
1221	1200	$\delta(CH)_{NBD}$	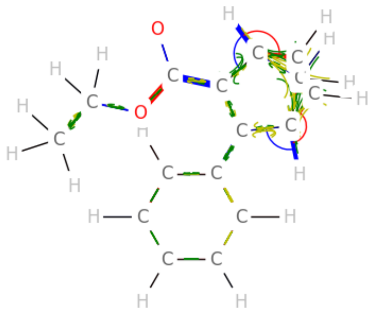

1238	1216	δ(CH)$_{NBD}$	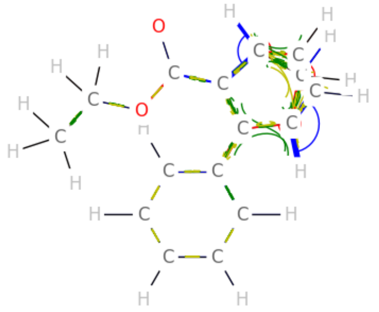
1252	1224	δ(CH)$_{NBD}$	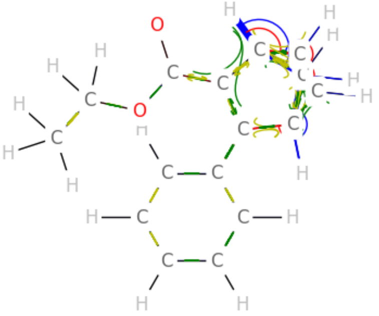
1280	1276	δ(CH)$_{NBD}$	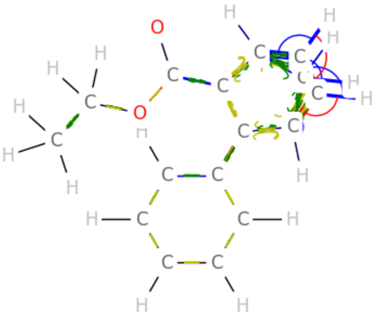
1295	1290	δ(CH)$_{phenyl}$, ν(CC)	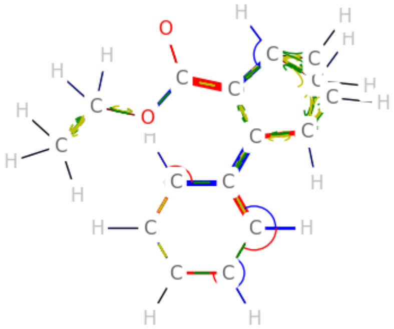
1331	1314	δ(CH)$_{phenyl}$	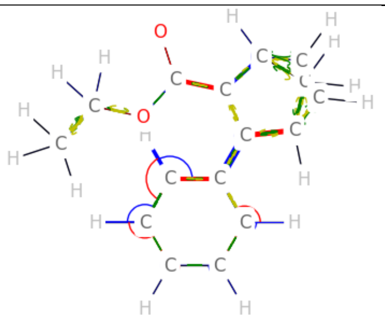

1370	1341	δ(CH)$_{ester}$	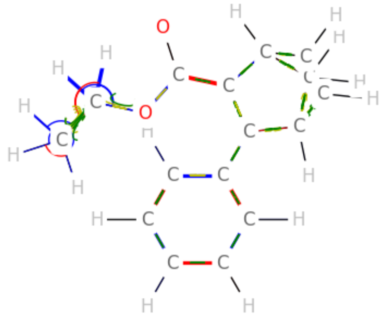
1392	1369	δ(CH)$_{ester}$	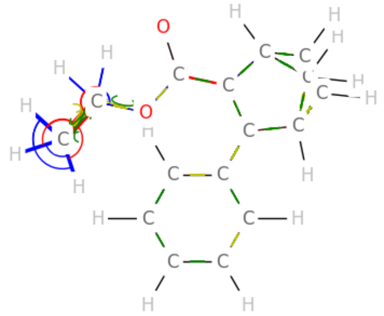
1445	1432	δ(CH)$_{phenyl}$	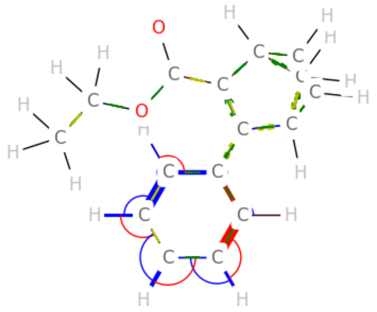
1477	1461	δ(CH)$_{ester}$	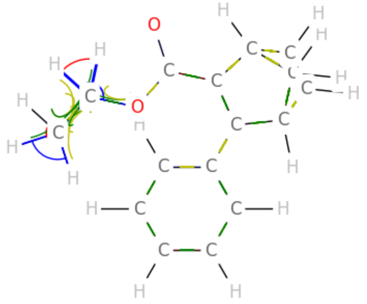
1492	1478	δ(CH)$_{phenyl}$, ν(CC)$_{phenyl}$	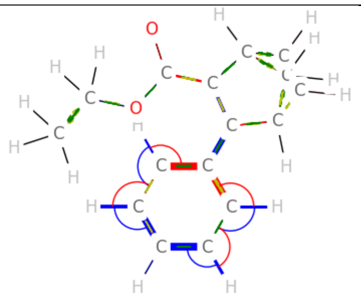

1559	1563	$\nu(CC)_{phenyl,}$ NBD	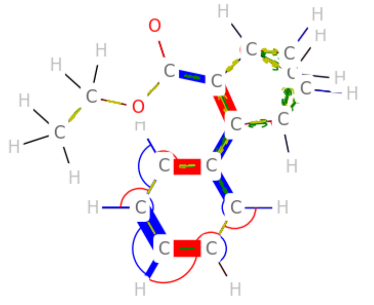
1574	1571	$\nu(CC)_{phenyl,}$ NBD	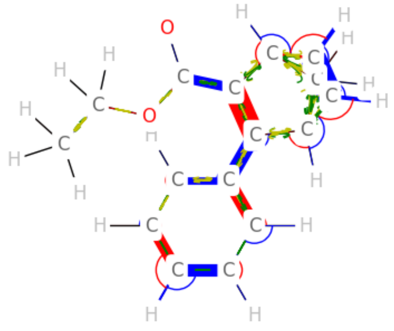
1596	1592	$\nu(CC)_{phenyl,}$ NBD	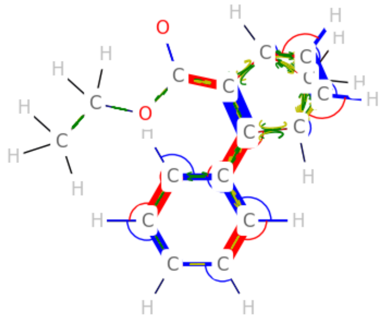
1611	1597	$\nu(CC)_{phenyl,}$ NBD	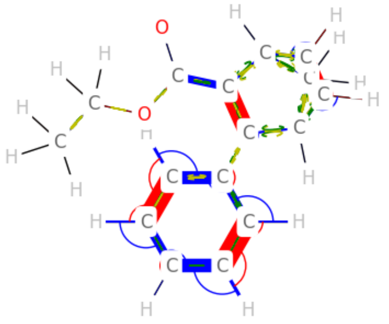
1693	1683	$\nu(CO)$	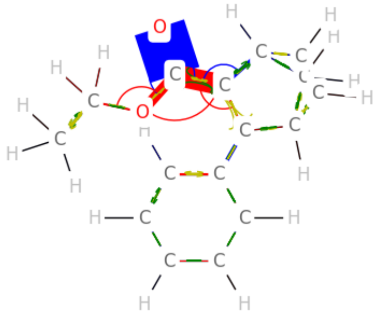

Table SI2: Peak assignment for QC1 based on transmission spectra and DFT calculations.

**Band positions in the transmission spectrum of QC1 [cm^{-1}]**	**Theoretical predicted band positions of QC1 by DFT [cm^{-1}]**	**Vibrational mode**	**2D representations of the vibrational modes**
1162	1134	$\nu(CH)_{ester}$, $\delta(CH)_{ester}$	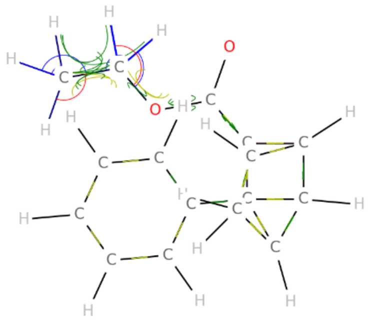
1187	1153	$\nu(CH)_{QC}$, $\delta(CH)_{QC}$	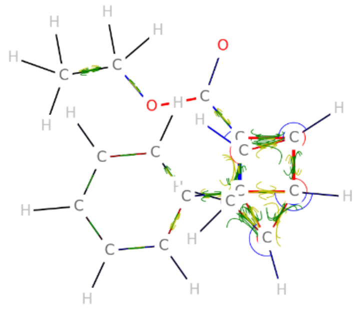
1219	1191	$\nu(CH)_{QC}$, $\delta(CH)_{QC}$	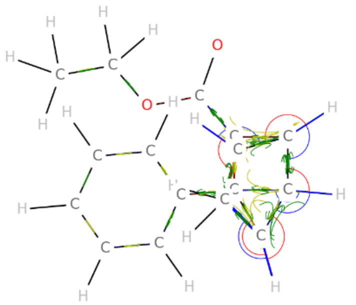
1236	1208	$\nu(CC)_{QC}$	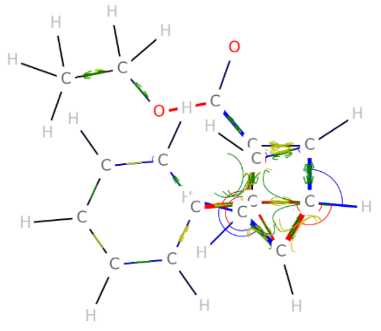

1253	1227	$\nu(CC)_{QC}$, $\delta(CH)_{QC}$	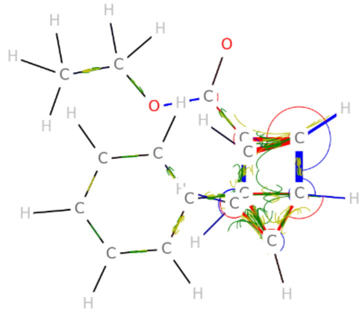
1297	1278	$\nu(CC)_{QC}$, $\delta(CH)_{QC}$	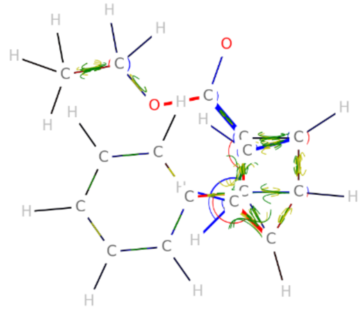
1340	1336	$\delta(CH)_{ester}$	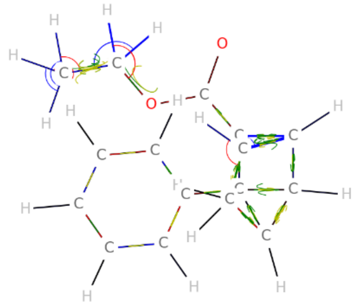
1377	1361	$\delta(CH)_{ester}$	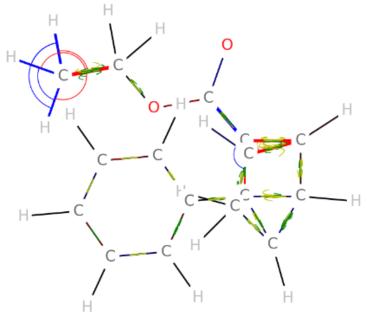
1396	1379	$\delta(CH)_{ester}$	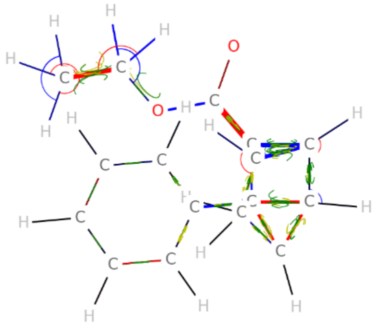

1408	1386	$\nu(CC)_{QC}$	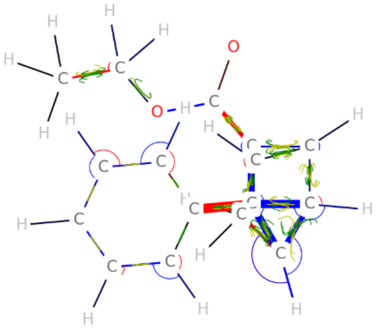
1445	1434	$\delta(CH)_{phenyl}$	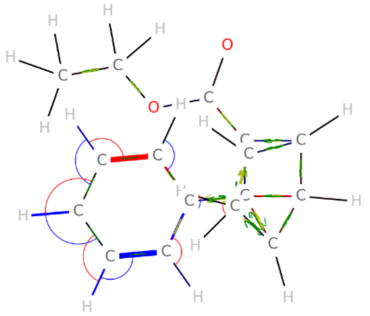
1454	1445	$\delta(CH)_{ester}$	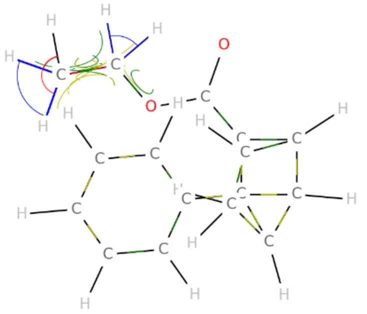
1475	1464	$\delta(CH)_{ester}$	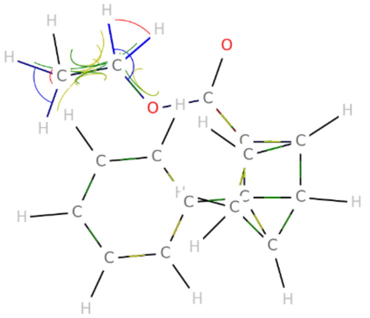
1499	1490	$\nu(CC)_{phenyl}$, $\delta(CH)_{phenyl}$	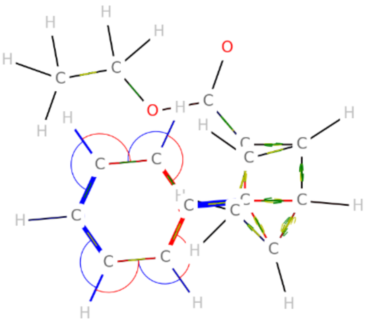

1576	1575	ν(CC)$_{phenyl}$	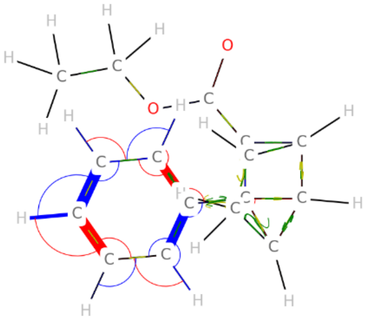
1601	1601	ν(CC)$_{phenyl}$	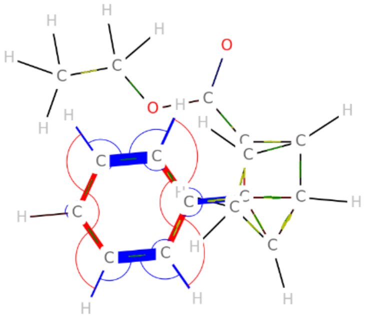
1692	1711	ν(CO)	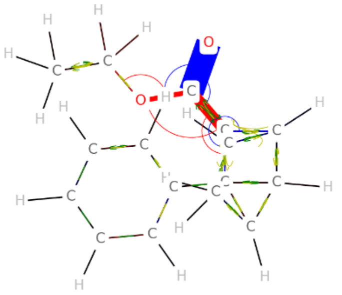

Table SI3: Peak assignment for the most intense peaks of NBD2 based on transmission spectra and DFT calculations.

**Band positions in the transmission spectrum of QC1 [cm^{-1}]**	**Theoretical predicted band positions of QC1 by DFT [cm^{-1}]**	**Vibrational mode**	**2D representations of the vibrational modes**
1151	1116	ν(CH)$_{phenyl}$	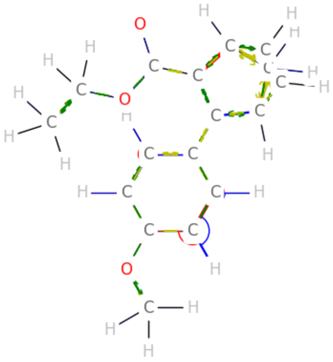

1175	1132	$\delta(CC)_{NBD}$, $\nu(CH)_{NBD}$, $\delta(CH)_{NBD}$	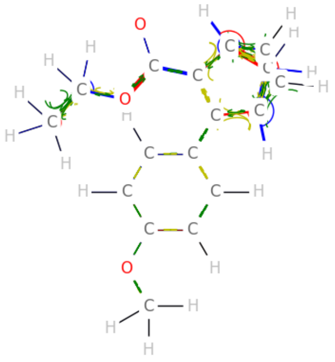
1190	1151	$\delta(CC)_{NBD}$, $\nu(CH)_{NBD}$, $\delta(CH)_{NBD}$	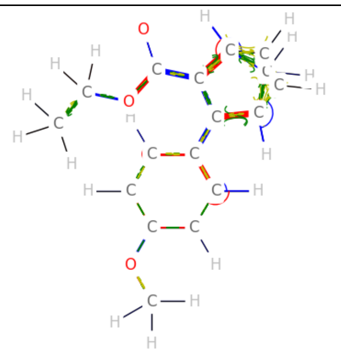
1220	1173	$\delta(CH)_{phenyl}$	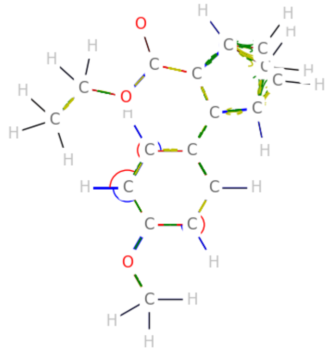
1236	1200	$\nu(CH)_{NBD}$, $\delta(CH)_{NBD}$	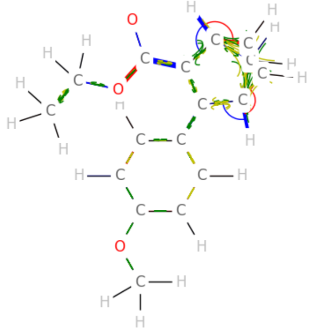

1253	1216	$\delta(CH)_{NBD}$	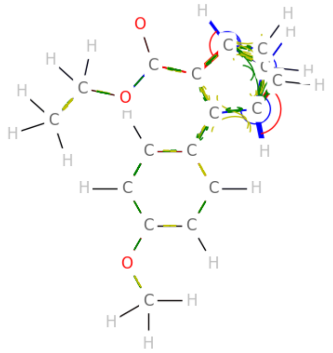
1295	1255	$\nu(COC)_{methoxy}$	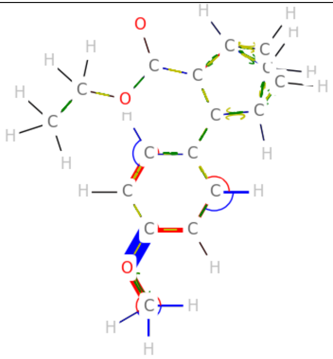
1309	1278	$\delta(CH)_{NBD}$	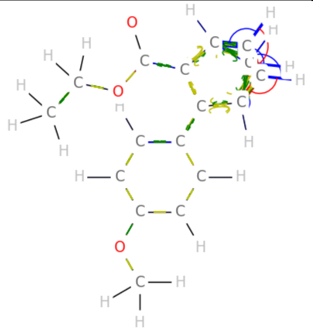
1331	1308	$\nu(CC)_{phenyl, NBD}$	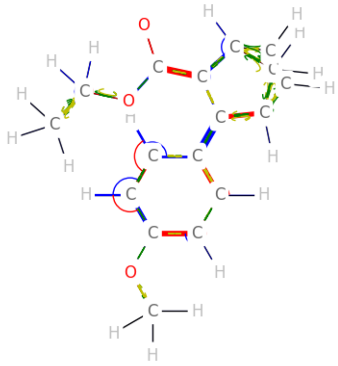

1369	1337	$\delta(CH)_{ester}$, $\nu(CH)_{ester}$, $\nu(CC)_{phenyl}$	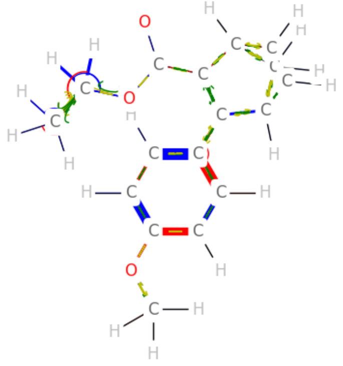
1369	1344	$\delta(CH)_{ester}$, $\nu(CH)_{ester}$	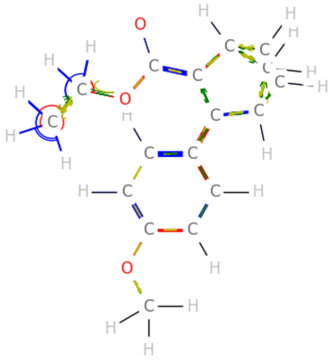
1391	1368	$\delta(CH)_{ester}$, $\nu(CH)_{ester}$	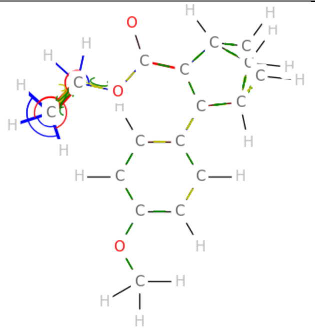
1417	1417	$\delta(CH)_{phenyl, methoxy}$, $\nu(CC)_{phenyl}$	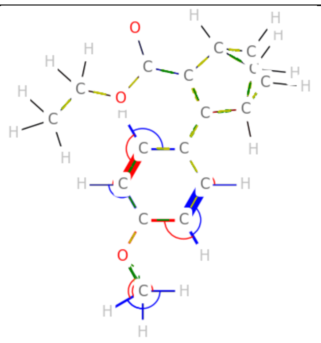

1443	1421	δ(CH)$_{methoxy}$	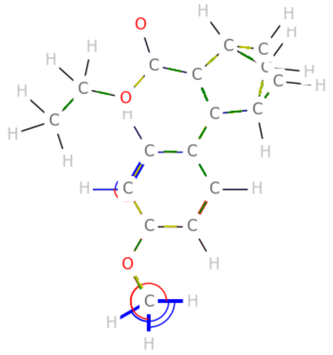
1455	1437	δ(CH)$_{NBD}$	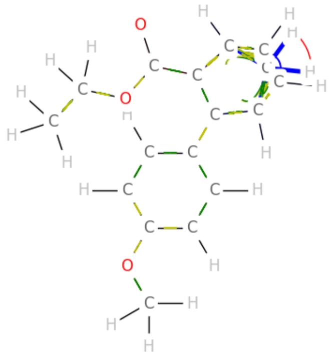
1464	1438	δ(CH)$_{methoxy}$	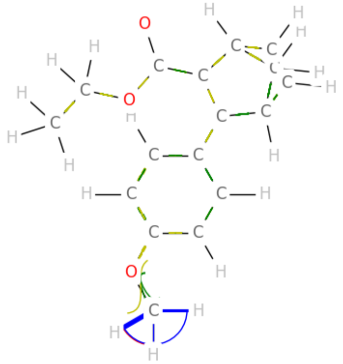
1464	1451	δ(CH)$_{methoxy}$	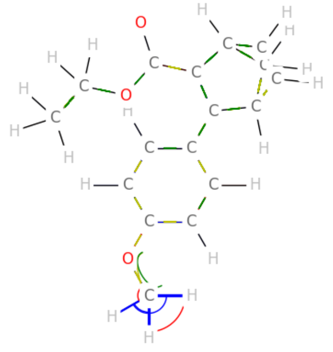

1477	1462	δ(CH)$_{ester}$	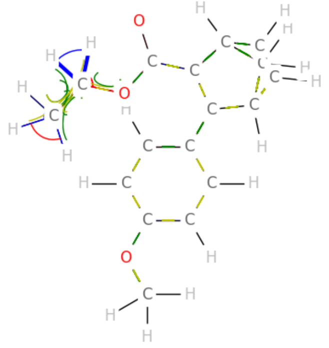
1509	1493	ν(CC)$_{phenyl}$, δ(CH)$_{phenyl}$	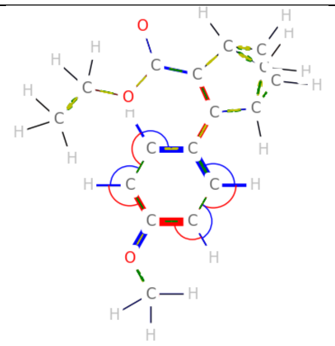
1557	1563	ν(CC)$_{phenyl, NBD}$	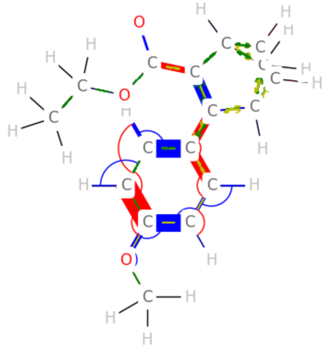
1572	1585	ν(CC)$_{phenyl, NBD}$, ν(CH)$_{NBD}$	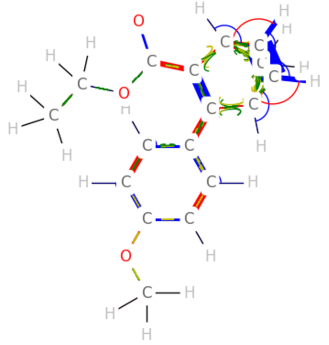

1605	1605	ν(CC)$_{phenyl, NBD}$	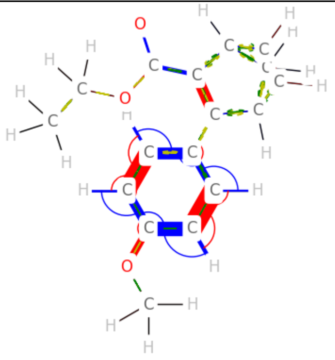
1698	1675	ν(CO)	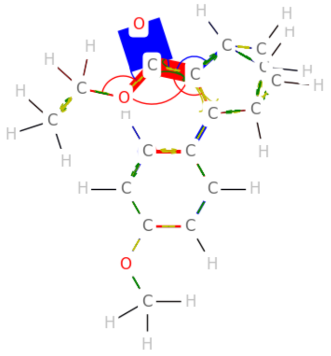

Table SI4: Peak assignment for QC2 based on transmission spectra and DFT calculations. .

**Band positions in the transmission spectrum of QC1 [cm^{-1}]**	**Theoretical predicted band positions of QC1 by DFT [cm^{-1}]**	**Vibrational mode**	**2D representations of the vibrational modes**
1162	1152	ν(CC)$_{QC}$, δ(CH)$_{QC}$	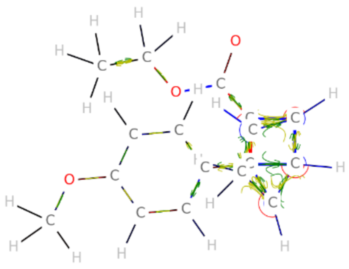

1180	1167	$\delta(CH)_{phenyl}$	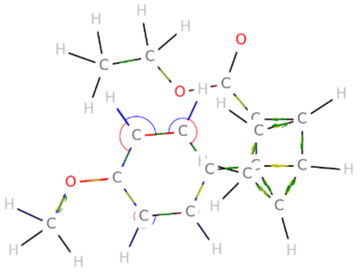
1209	1208	$\nu(CC)_{QC}$, $\delta(CH)_{QC}$	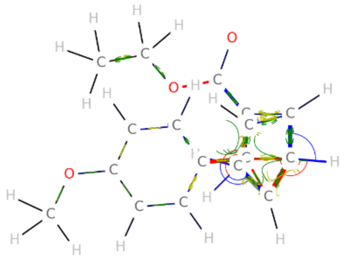
1221	1226	$\nu(CC)_{QC}$, $\delta(CH)_{QC}$	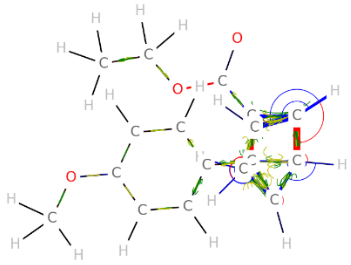
1247	1244	$\nu(COC)_{methoxy}$	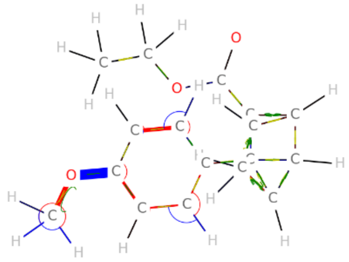
1299	1277	$\nu(CC)_{QC}$	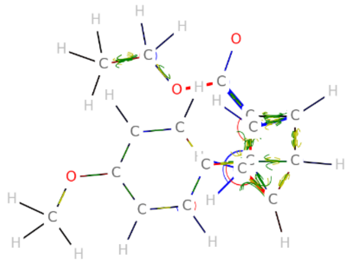

1341	1333	ν(CC)$_{phenyl}$	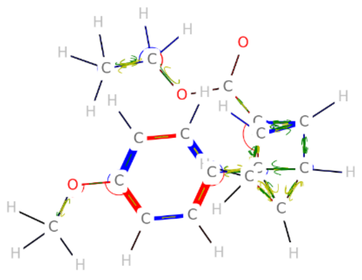
1379	1362	δ(CH)$_{ester}$	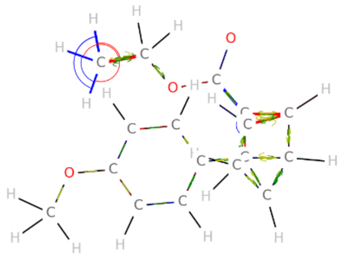
1399	1379	δ(CH)$_{ester}$, ν(CC)$_{QC,ester}$	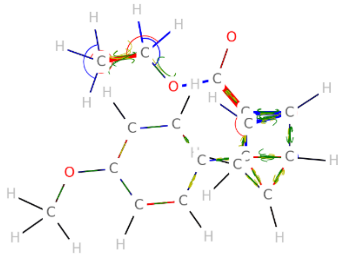
1399	1388	ν(CC)$_{QC}$	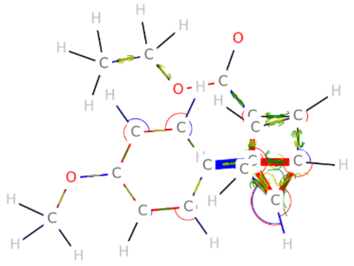
1416	1410	δ(CH)$_{phenyl}$, ν(CC)$_{phenyl}$	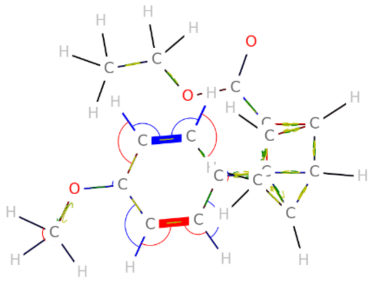

1444	1437	δ(CH)$_{methoxy}$	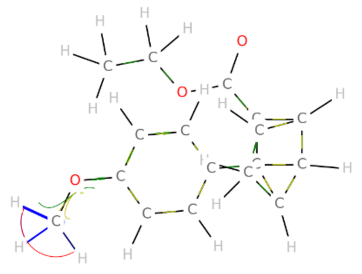
1465	1453	δ(CH)$_{methoxy}$	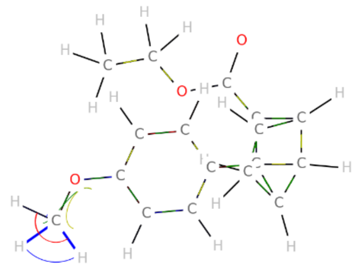
1518	1505	ν(CC)$_{phenyl}$, δ(CH)$_{phenyl}$	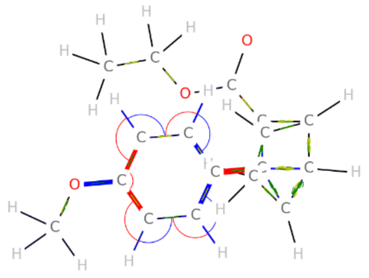
1578	1568	ν(CC)$_{phenyl}$	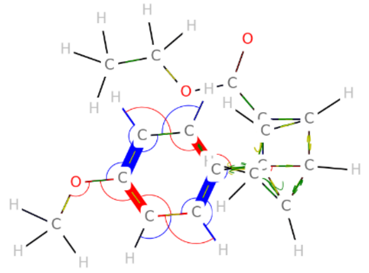
1606	1611	ν(CC)$_{phenyl}$	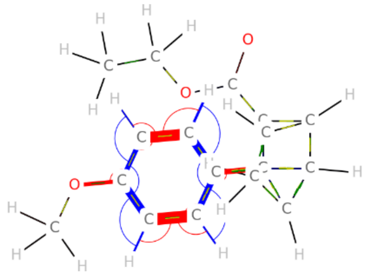

1715	1710	ν(CO)	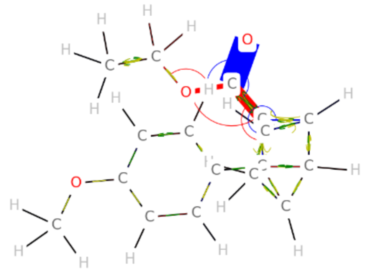

5. Photochemical conversion

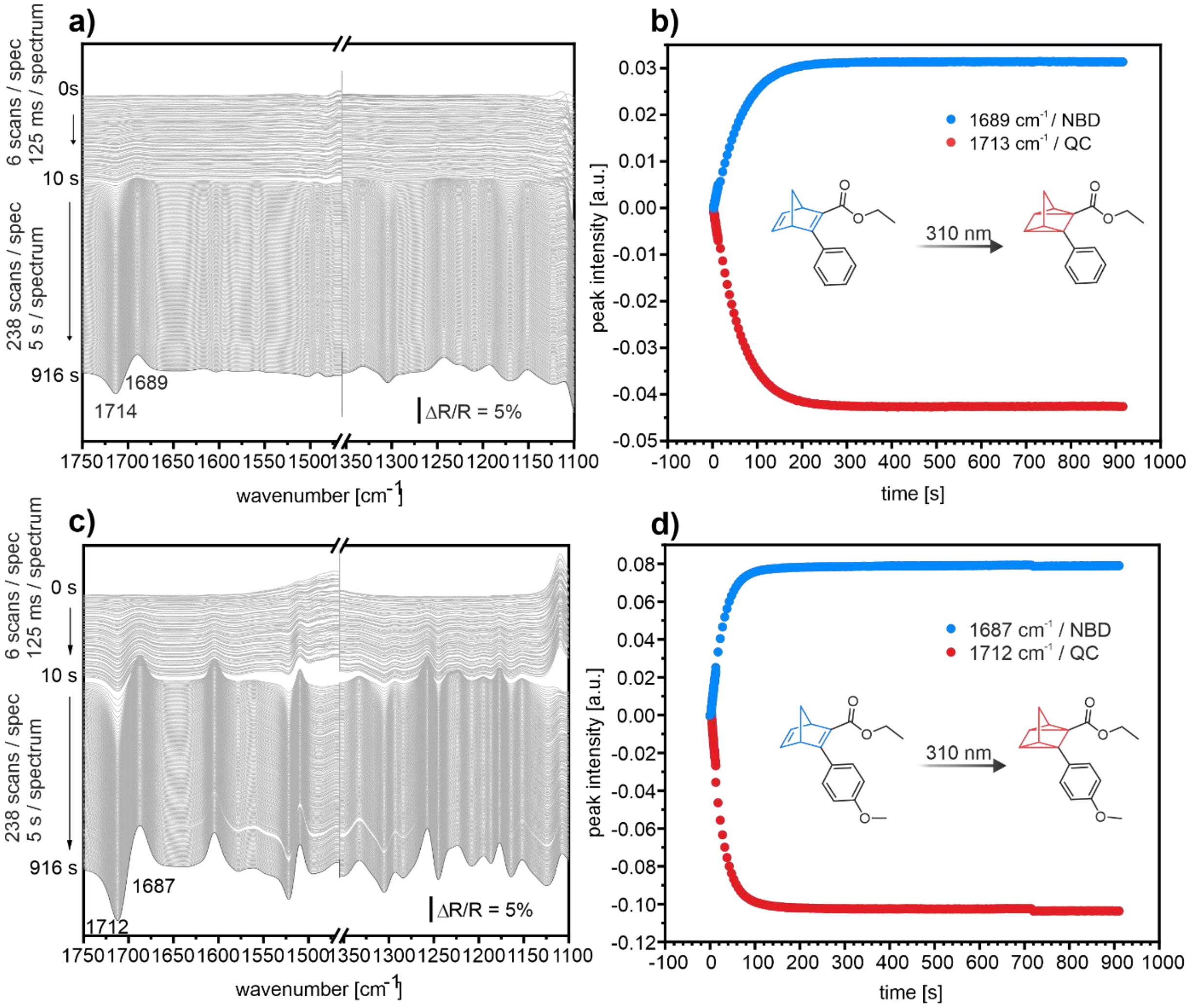

Figure SI5: (a) Time resolved IRRA spectra of 10 mM NBD1 in MeCN during irradiation at 310 nm; (b) band intensities of the ν(CO) vibrations during irradiation; (c) time resolved IRRA spectra of 10 mM NBD2 in MeCN during irradiation at 310 nm; (d) intensities of the ν(CO) band vs. time.

In order to investigate the photochemical conversion, we formed a thin layer in our PEC-IRRAS setup.[3] We used a HOPG crystal as working electrode and 10 mM NBD1/NBD2 dissolved in 0.1 M TBAP in MeCN as reactant. We irradiated the thin layer with a LED λ_{max} = 310 nm and recorded IRRA spectra simultaneously at OCP. In the first 10 s, we recorded time resolved spectra with an acquisition time of 125 ms/spectrum. Afterwards, we increased the acquisition time to 5 s/spectrum. In total, we irradiated for 916 s. The resulting spectra of NBD1 are depicted in Figure SI5a. Positive (pointing upwards) and negative (pointing downwards) bands form over time, as indicated exemplarily by the $\nu(CO)_{carbonyl}$ bands at 1689 cm^{-1} (NBD) and 1714 cm^{-1} (QC), respectively. The low acquisition time increases noise in the spectra. For this reason, we used the peaks with the highest intensity and not the spectroscopic marker at ~1500 cm^{-1} for this plot. Note that positive bands indicate consumed and negative bands

indicate formed species. Consequently, this indicates photochemical conversion from NBD to QC. Figure SI5b shows the peak intensity of these two bands depending on the time. We observe a saturation after approximately 200 s. Figure SI5c shows the IR spectra of NBD2 during irradiation. We observe the formation of positive (1687 cm^{-1}, ν(CO), NBD) and negative (1712 cm^{-1}, $ν(CO)_{carbonyl}$, QC) bands, again. In Figure SI5d the corresponding peak intensities of the carbonyl bands are depicted. Saturation is achieved by around half of the time, namely 100 s. Note that no spacer was used and the layer thickness may vary. This may additionally affect the conversion times.

6. **Calculated energies**

Table S51: Calculated energies of both NBDs and QCs.

	**Gas phase**	**In MeCN**
**Compound**	**Total energy [H]**	**Total energy [H]**
NBD1	-769.077448	-769.089026
$NBD1^{+\cdot}$	-768.810107	-768.880441
QC1	-769.047375	-769.060476
$QC1^{+\cdot}$	-768.792203	-768.865649
NBD2	-883.523948	-883.537757
$NBD2^{+\cdot}$	-883.272905	-883.340043
QC2	-883.491522	-883.506735
$QC2^{+\cdot}$	-883.251623	-883.321441

Table S51 shows the energies of the investigated compounds in gas phase and in MeCN, calculated by DFT. We observe that the difference between $QC1^{+\cdot}$ and QC1 is higher than the difference of $QC2^{+\cdot}$ and QC2. This indicates, that the oxidation potential of QC2 is lower than the one of QC1. The data also indicate, that the oxidation is stabilized in MeCN in comparison to the gas phase.

7. **Quantitative analysis**

The concentrations of NBD1/QC1 and NBD2/QC2 were determined according to Beer-Lamberts law

$$\mathrm{A} = \log\frac{I_0}{I} = \varepsilon \cdot d \cdot c$$

Our spectra are recorded as difference spectra, which means that we divide the recorded spectrum by the background spectrum. From this, the absorbance was calculated by

$$\mathrm{A} = -\log\frac{I}{I_0} = \varepsilon \cdot d \cdot c$$

with A = absorbance at 1493 cm^{-1} (NBD1), 1510 cm^{-1} (NBD2), 1501 cm^{-1} (QC1), and 1519 cm^{-1} (QC2), the extinction coefficient ε and the layer thickness d.

The concentration of the NBD is known to be 10 mM. By the absence of the peaks assigned to the NBD derivates after irradiation in the single channel spectrum, we determined that the compound is fully converted. By ^{1}H-NMR spectroscopy, we confirmed that no side products are formed upon irradiation in MeCN and all compound is converted to QC. Therefore, the QC concentration after irradiation was normalized with respect to the NBD consumption.

8. **Band assignment of NBD1 and QC1 in the IR spectra**

Figure SI6 shows the full IR spectrum of the electrochemically triggered back-conversion of the NBD1/QC1 system on HOPG (discussed in detail in the main text). After irradiation, characteristic bands appear. Positive bands, which we assign to NBD1, are present at 1690 cm^{-1} (ν(CO)), 1595 cm^{-1} ($\nu(C{=}C)_{phenyl,\ NBD}$), 1558 cm^{-1} ($\nu(C{=}C)_{phenyl,\ NBD}$), 1493 cm^{-1} ($\delta(CH)_{phenyl}$), 1332 cm^{-1} ($\delta(CH)_{phenyl}$), and 1243 cm^{-1} ($\delta(CH)_{NBD}$). We observe negative bands at 1712 cm^{-1} (ν(CO)), 1603 cm^{-1} ($\nu(CC)_{phenyl}$), 1500 cm^{-1} ($\delta(CH)_{phenyl}$) and 1303 cm^{-1} ($\nu(CC)_{QC,\ ester}$), which we assign to the formed QC1. In the shown difference spectra, positive (blue, upwards oriented) bands correspond to species which are consumed while negative (red, downwards oriented) bands are related to the formation of a species with respect to the measured background. As mentioned in the main text, the ν(CO) band of NBD1 and QC1 form a s-shaped band upon irradiation although they are overlapping (see Figure 4). This can be explained by the change of the shape of this band (see Figure SI7). The shoulder of the NBD1 band vanishes, while the

peak maximum is higher for QC1. From the resulting bands in the difference spectrum, it is possible to follow the reaction, but their overlap does not allow a quantitative analysis. For this reason, the ν(CC) band was used to calculate the concentrations. Additional features at 1450 cm^{-1}, 2250 cm^{-1}, 2413 cm^{-1}, and 2627 cm^{-1} are associated with the solvent MeCN.[4]

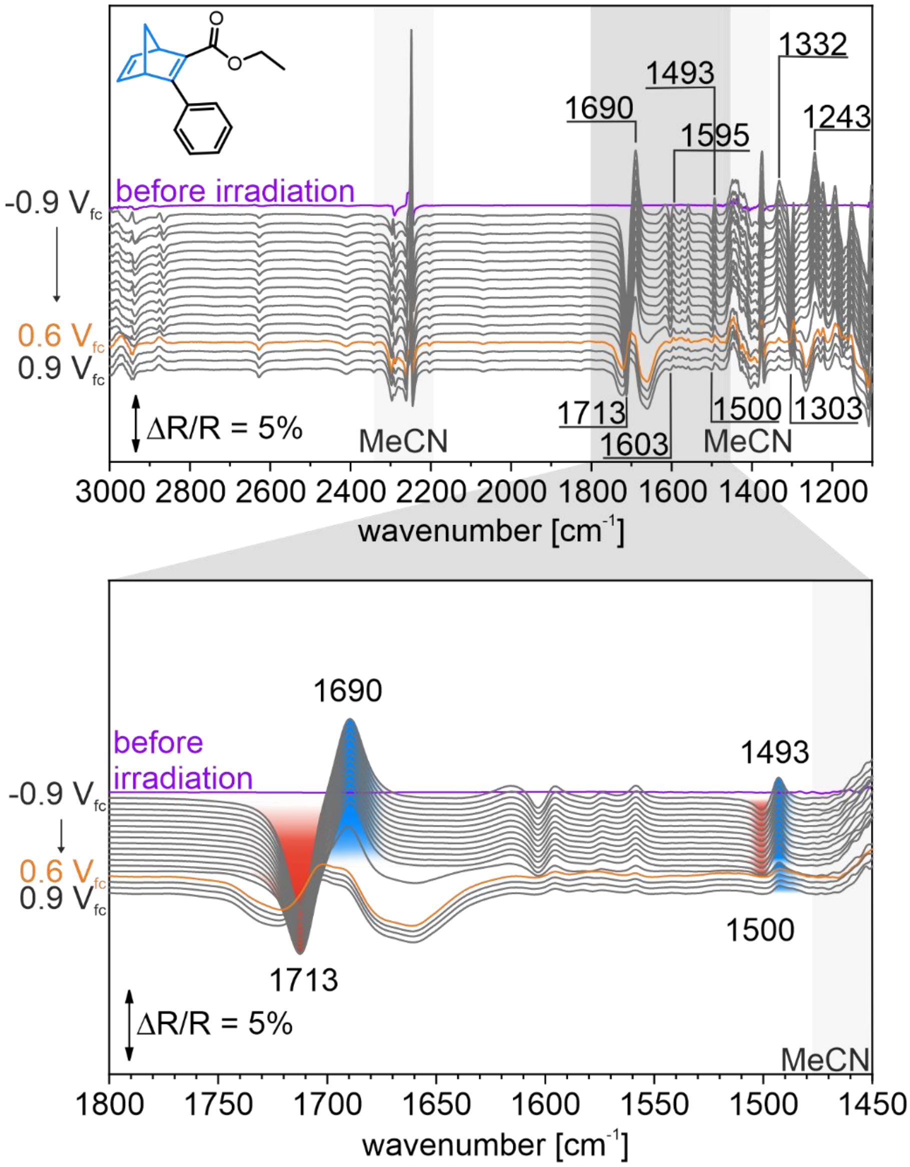

Figure SI6: Electrochemically triggered back-conversion of NBD1. (a) IRRA-spectra of the photochemical conversion and electrochemical back-conversion in the NBD1/QC1 system on HOPG; (b) *ν*(CO) and *ν*(CC) region as a function of the electrode potential. The reference spectra were taken at -0.9 V_{fc}.

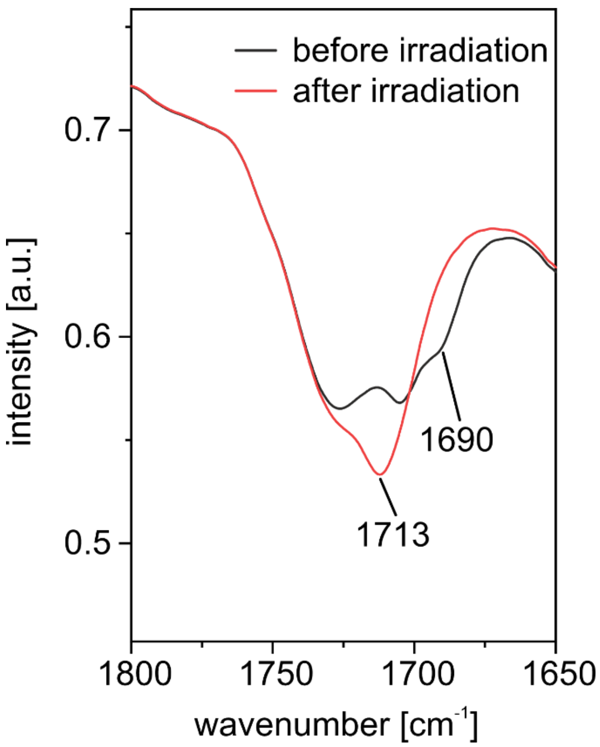

Figure SI7: Single channel spectra of the ν(CO) band before (NBD1) and after irradiation (QC1) at -0.9 V_{fc}.

9. **Band assignment of NBD2 and QC2 in the IR spectra**

Figure SI8 shows the full spectrum of the electrochemically triggered back-conversion of the NBD2/QC2 system on HOPG provided in Figure 6. After irradiation, positive bands at 1686 cm^{-1} (ν(CO)), 1605 cm^{-1} (ν(CC, CO)$_{phenyl}$), 1509 cm^{-1} (ν(CC, CO)$_{phenyl, NBD}$), 1332 cm^{-1} (δ(CH)$_{phenyl}$), 1255 cm^{-1} (δ(CH)$_{NBD}$) and negative bands at 1711 cm^{-1} (ν(CO)), 1579 cm^{-1} (ν(CC, CO)$_{phenyl}$), 1519 cm^{-1} (δ(CH)$_{phenyl}$, 1305 cm^{-1} (δ(CH)$_{QC}$), and 1255 cm^{-1} (ν(COC)$_{methoxy}$) indicate the conversion from NBD2 to QC2. Additional bands are assigned to MeCN.

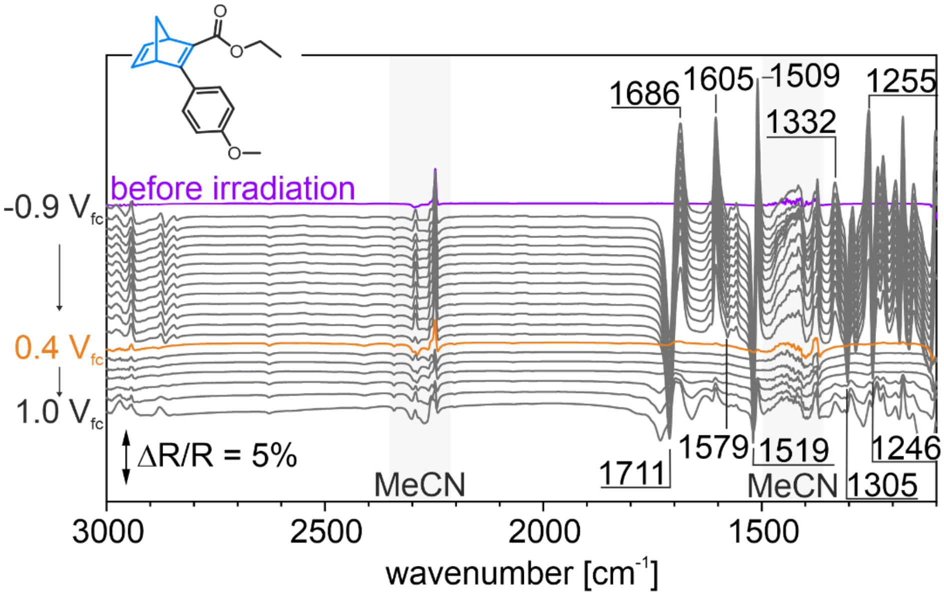

Figure SI8: Electrochemically triggered back-conversion of NBD2. IRRA-spectra of the photochemical conversion and electrochemical back-conversion in the NBD2/QC2 system on HOPG.

10. **Number of converted QC2 molecules per electron**

In order to estimate roughly how many QC2 molecules are converted per electron, we compare the measured charge from the DPV of QC1 and QC2. We assume that the electrode surface area is identical and that at the surface all QC is consumed (conversion or decomposition) due to diffusion limitations. For QC1, we know from the IRRAS data, that 0.018 C correspond to 40% decomposition. We assume that the oxidative decomposition during the reconversion of QC1 is a one-electron process. From this we conclude, that 0.018 C is necessary to convert 40% QC1 to its decomposition product. In contrast to this, 0.002 C charge is observed for the back-conversion of QC2. If we compare these two charge values, it follows that with 1/9 of the charge of the decomposition of QC1, (see main text) 100% of the QC2 is back-converted. We concluded that 1 electron converts:

$$\frac{ratio\ converted\ compound}{ratio\ charge} = \frac{compound}{charge} = \frac{(\frac{1}{0.4})}{(\frac{0.002}{0.018})} = \frac{1}{0.4} \cdot 9 = 22.5$$
$$\approx 20\ \frac{QC2\ molecules}{1\ electron}$$

From this and a storage capacity of 87.4 $kJmol^{-1}$[1] we estimate, that 1 C electric charge releases 0.017 kJ heat. Considering the capacity of a battery, 1 Ah is needed to release 63 kJ heat.

11. **Cyclability of NBD2/QC2**

In order to investigate the reversibility of the storage process, we performed 100 storage and release cycles. Every cycle included irradiation at -0.9 V_{fc} (photochemical conversion, 150 s) and energy release after a potential jump to 0.5 V_{fc} (electrocatalytically triggered back-conversion, 60 s) as illustrated in Figure SI9a. Between each step, we measured spectra (S_M) at -0.9 V_{fc}. Note that mass exchange between the thin-layer and the bulk solution occurs on the timescale of several hours only.[5,6] All spectra refer to the reference spectrum (S_R) measured before the 100 cycles at -0.9 V_{fc}. In Figure SI9b, we show exemplarily the resulting spectra in the region of the ν(CO) band after cycle 1, cycle 10, cycle 50, and cycle 100. After irradiation (black spectra), we observe a positive band at 1686 cm^{-1} and a negative band 1712 cm^{-1}, characteristic for the NBD2 to QC2 isomerisation. The intensity of the band at 1686 cm^{-1} remains identical over 100 cycles, indicating that NBD is converted quantitatively during the

photochemical process within 100 conversion cycles. The intensity of the band at 1712 cm^{-1}, however, decreases over the course of the 100 cycles, indicating some decomposition during the electrochemically triggered back-conversion. After the potential jump to 0.5 V_{fc}, the bands vanish after the first cycle. After cycle 10, we observe a small feature remaining at 1686 cm^{-1} and a new negative band at 1736 cm^{-1}, which we assign to decomposition products. The intensity of the remaining band and the new band increase with ongoing cycling, which confirms the partial decomposition during back-conversion. By comparing the intensities, it is possible to quantify the fraction of decomposition within the 100 conversion cycles by the Beer-Lambert law (see Figure SI9c). To minimize the influence of the background, we used the spectrum before photoconversion as background, respectively. The determined decomposition after 100 conversion cycles is only 30%, which is comparable to the NBD/QC system which reached the highest reversibility so far using the electrochemically triggered energy release.[7]

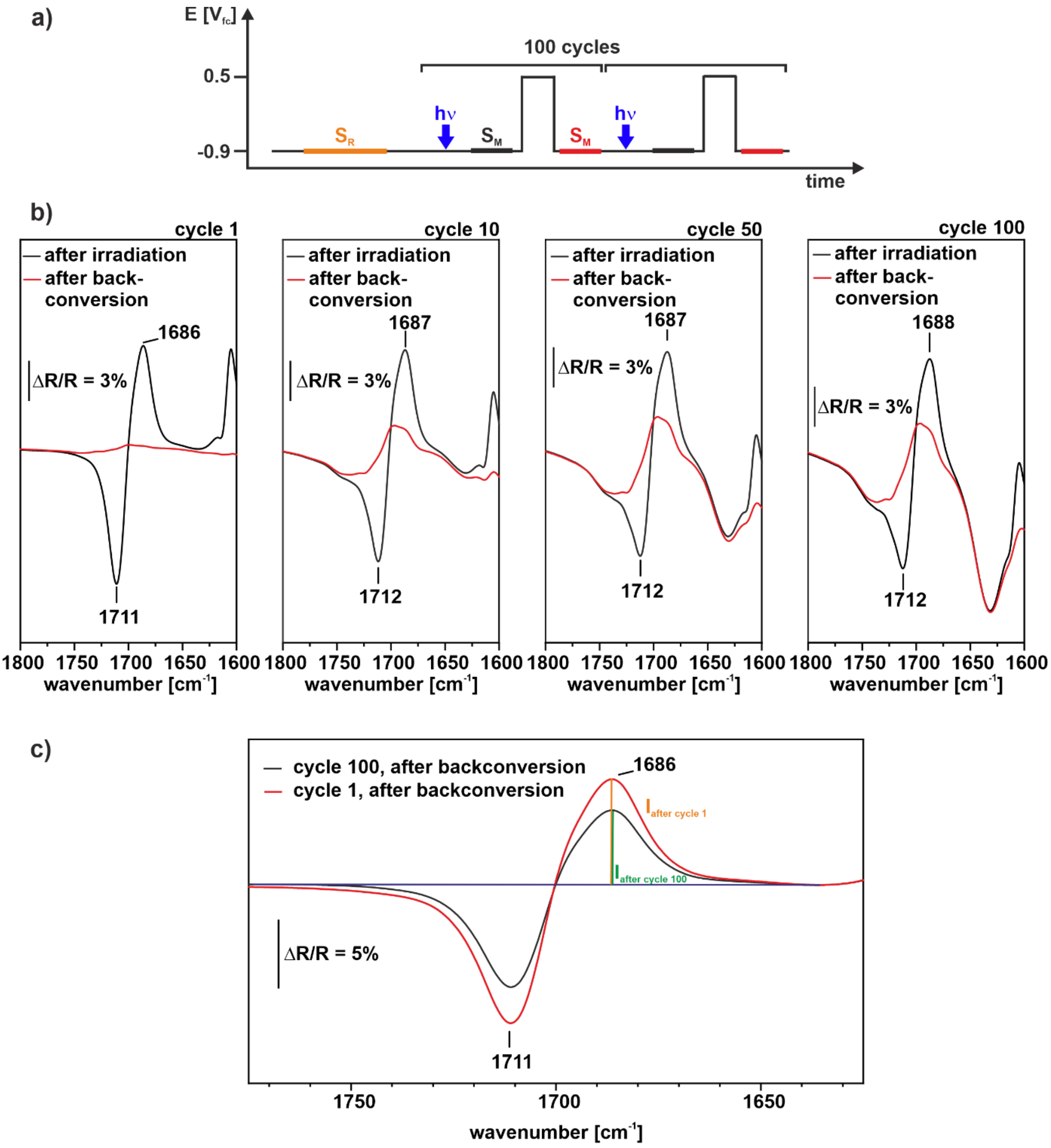

Figure SI9: Stability test of the electrochemically triggered NBD2/QC2 storage system; (a) experimental procedure covering 100 conversion cycles; (b) selected PEC-IRRA spectra (cycle 1, 10, 50, and 100) in the region of the ν(CO) band after irradiation (black) and after electrochemically triggered back-conversion (red) (reference spectrum recorded at -0.9 V_{fc}); (c) spectrum after photoconversion cycle 1 and cycle 100 with the spectrum recorded before as background.

12. **Calculated Energies with B3LYP**

To check the dependence of the calculated energies on the choice of the exchange-correlation functional, we studied the oxidative reaction channel for the two different NBD/QC derivatives with calculations using B3LYP exchange-correlation functional. The energies of the considered QC, $QC^{+\cdot}$, $NBD^{+\cdot}$ and NBD compounds relative to the QC molecule are depicted in Figure SI10. For QC1 (left), we obtained an ionization energy of 7.0 eV (with the PBE exchange correlation, we obtained 6.9 eV). For QC2, this energy decreases to 6.6 eV (with PBE we obtained 6.5 eV). In conclusion, the ionization energies change only negligible upon change of the density-functional and the observed trend, the decreased ionization energy of QC2 in comparison to QC1, is independent of the used density functional. Also, the energy difference is very small.

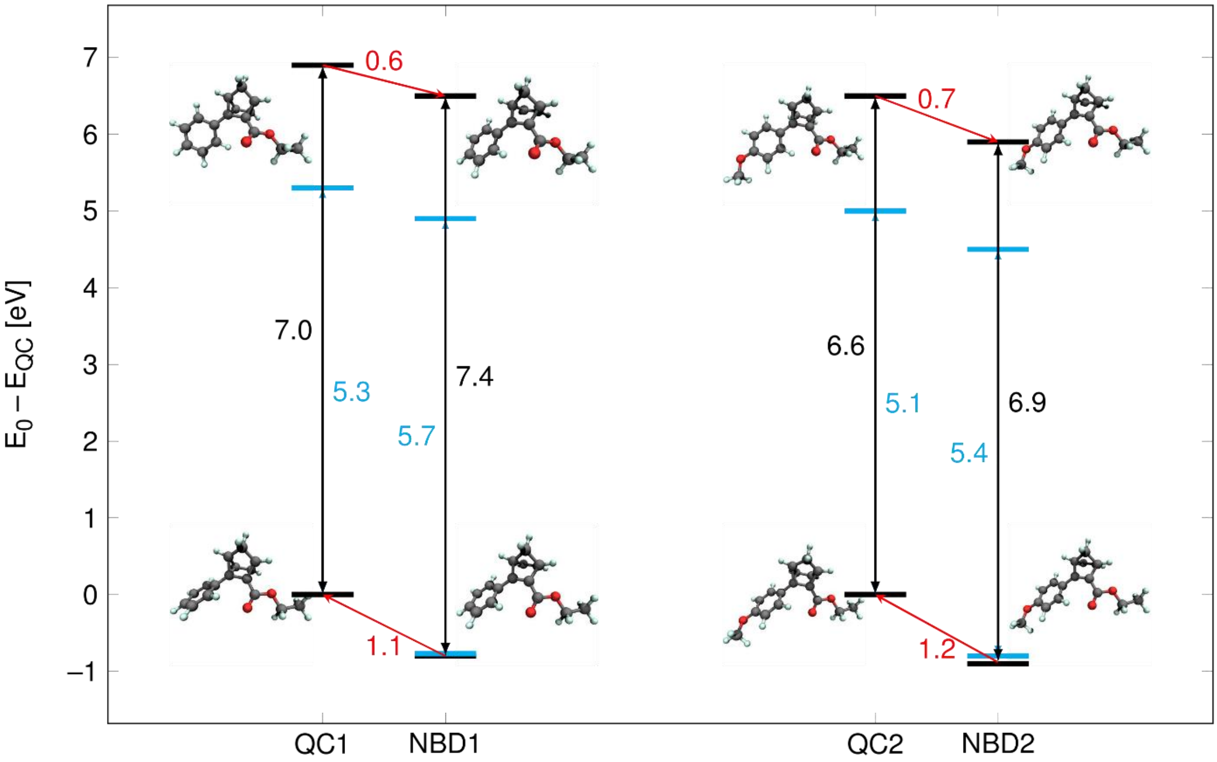

Figure SI10: Energies of the considered QC, $QC^{+\cdot}$, $NBD^{+\cdot}$, and NBD compounds relative to QC for the derivatives NBD1/QC1 and NBD2/QC2 as derived from DFT calculations with the B3LYP exchange-correlation functional (see text for details). Black: calculations in gas phase; blue: Calculations considering the COSMO solvent model.

13. **Visualization of molecular orbitals**

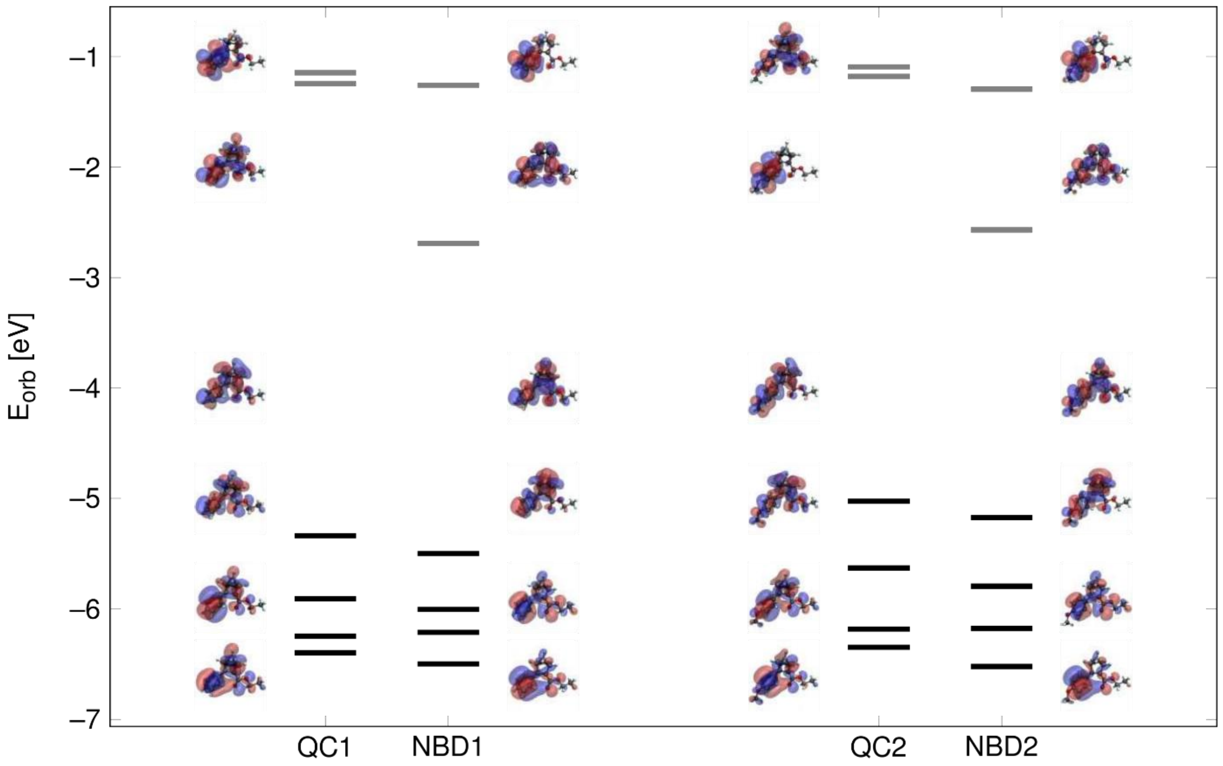

Figure SI11: Eigenvalues and contour plots of the four energetically highest occupied and the two energetically lowest unoccupied orbitals of NBD1, QC1, NBD2, and QC2 calculated with the PBE functional including the COSMO model (contour values ± 0.01 a.u.).

14. **References**

[1] P. Lorenz, T. Luchs, A. Hirsch, *Chem. - A Eur. J.* **2021**, *27*, 4993–5002.

[2] M. Laurin, *J. Chem. Educ.* **2013**, *90*, 944–946.

[3] O. Brummel, F. Waidhas, U. Bauer, Y. Wu, S. Bochmann, H. P. Steinrück, C. Papp, J. Bachmann, J. Libuda, *J. Phys. Chem. Lett.* **2017**, *8*, 2819–2825.

[4] E. L. Pace, L. J. Noe, *J. Chem. Phys.* **1968**, *49*, 5317–5325.

[5] T. Iwasita, F. C. Nart, *Prog. Surf. Sci.* **1997**, *55*, 271–340.

[6] F. Waidhas, M. Jevric, L. Fromm, M. Bertram, A. Görling, K. Moth-Poulsen, O. Brummel, J. Libuda, *Nano Energy* **2019**, *63*, 103872.

[7] F. Waidhas, M. Jevric, M. Bosch, T. Yang, E. Franz, Z. Liu, J. Bachmann, K. Moth-Poulsen, O. Brummel, J. Libuda, *J. Mater. Chem. A* **2020**, *8*, 15658–15664.
